# Rational analysis of data from LC-MS/MS: new insights in acylcarnitines as biomarkers for brain disorders or neurotoxicity

**DOI:** 10.3389/fphar.2024.1441755

**Published:** 2024-08-22

**Authors:** Li Chen, Ruiqin Zhu, Yaxing Ma, Chuixiu Huang, Xiantao Shen

**Affiliations:** ^1^ Key Laboratory of Environment and Health, Ministry of Education & Ministry of Environmental Protection and State Key Laboratory of Environmental Health (Incubation), School of Public Health, Tongji Medical College, Huazhong University of Science and Technology, Wuhan, China; ^2^ Department of Forensic Medicine, Huazhong University of Science and Technology, Wuhan, China

**Keywords:** phenobarbital poisoning, biomarker screening, data analysis, acylcarnitines, two strategies

## Abstract

**Objective:**

LC-MS/MS-based metabolomics is an important tool for studying disease-related biomarkers. Conventionally, different strategies have been used to screen biomarkers. However, many studies for biomarker screening by different strategies have ignored the dose-response relationship between the biomarker level and exposure level, and no relevant studies have described and compared different strategies in detail. Phenobarbital (PHB) which belongs to the barbiturates, was selected as the typical representative of neurotoxins. Acylcarnitines have been promising candidates for diagnostic biomarkers for several neurological disorders and neurotoxicity. In this work, we aimed to use an acute PHB poisoning animal model to clarify PHB poisoning effects on plasma and brain acylcarnitine changes and how to rationally analyze data from LC-MS/MS.

**Methods:**

The acylcarnitine profiles in plasma and brain regions in an actuate PHB poisoning animal model were utilized. The dose-response relationship between plasma PHB and carnitine and acylcarnitines (CARs) in plasma and brain were assessed by the variance analysis trend test and Spearman’s rank correlation test. In different strategies, principal component analysis (PCA) and partial least squares discriminant analysis (OPLS-DA) screened the differential CARs, variable importance plots (VIPs) were utilized to select putative biomarkers for PHB-induced toxicity, and receiver operating characteristic (ROC) curve analysis then illustrated the reliability of biomarkers.

**Results:**

Under the first strategy, 14 potential toxicity biomarkers were obtained including eight downregulated CARs with AUC >0.8. Under the second strategy, 11 potential toxicity biomarkers were obtained containing five downregulated CARs with AUC >0.8. Only when the dose-response relationship was fully considered, different strategies screen for the same biomarkers (plasma acetyl-carnitine (C2) and plasma decanoyl-carnitine (C10)), which indicated plasma acylcarnitines might serve as toxicity biomarkers. In addition, the plasma CAR level changes showed differences from brain CAR level changes, and correlations between plasma CARs and their brain counterparts were weak.

**Conclusion:**

We found that plasma C2 and C10 might serve as toxicity biomarkers for PHB poisoning disorders, and PHB poisoning effects on changes in plasma CARs may not be fully representative of changes in brain CARs.

## 1 Introduction

The term “biomarker”, which usually refers to a broad sign of genetic or epigenetic cellular, biochemical, and molecular compounds in biological samples (e.g., plasma and tissues), is an indicator of a typical disease. Hence, biomarker discovery is a key step in clinical technology aiming at its applications in early cancer diagnosis, treatment evaluation and prognosis. In biomarker discovery studies, experiments using more human samples or fewer animal model samples were often performed (Gao, 2015). The biomarkers were always recognized using two strategies. In the first strategy, the selection of only one concentration group (animal model samples) of interest compared to a control group for biomarker screening was usually utilized (Ezaki et al., 2017). In the second strategy, a comparison of the healthy controls and cases (one combined poisoning group as poisoned cases in this study) was usually used to screen the differential metabolites (clinical or epidemiologic study) (Motsinger-Reif et al., 2013; Varma et al., 2018). As a principle, a satisfactory biomarker requires that the biomarker level possesses a dose-response relationship with the level of exposure, a comprehensive description and comparison between these two strategies (combining the biological samples in a combined poisoning group or selecting a typical dose group) has therefore been a great challenge to indicate their rationality in biomarker discovery (Huggett, 1992; Depledge et al., 1993; Svendsen et al., 2004; Ristic-Medic et al., 2014; Hong et al., 2017).

Synaptic energy state and mitochondrial dysfunction play important roles in various brain pathologies. As natural derivatives of carnitine, acylcarnitines can enhance brain energy metabolism since they fulfilled a neuroprotective effect role in the central nervous system, such as lipids synthesis (Ricciolini et al., 1998), genes and proteins regulation (Calabrese et al., 2004; Traina et al., 2004; Chiechio et al., 2006), antioxidant activity enhancement (Calabrese et al., 2006; Poon et al., 2006), and cholinergic neurotransmission improvement (Fariello et al., 1988; Sershen et al., 1991; Ando et al., 2001; Nalecz et al., 2004). Therefore, acylcarnitines have been promising candidates for diagnostic biomarkers for several neurological disorders and neurotoxicity. Generally, there were two kinds of acylcarnitines have been evaluated as biomarkers for neurological disorders. In the literature, acylcarnitines in plasma have been widely reported as biomarkers with evidence of neurological disorders and neurotoxicity (Ibáñez et al., 2012; Álvarez-Sánchez et al., 2022). For example, a Chinese male cohort study (with 95 healthy participants) also showed that people with high dioxin exposure levels were at potential health risks of inflammation, liver and cardiovascular, and acylcarnitines were identified as the potential biomarkers for dioxin toxicity (Liang et al., 2020). Except for acylcarnitines in plasma, acylcarnitines in brain regions were also reported as biomarkers for brain disorders. For example, by using matrix-assisted laser desorption/ionization-MS imaging, acylcarnitines were proven to have great potential in understanding how people who sustained a traumatic brain injury during their lifetime develop Parkinson in later stages of life (Mallah et al., 2019).

In order to better understand the rationality between the strategy using a selected dose group and the strategy using the combined poisoning group in biomarker discovery, the acylcarnitine profiles in the plasma and the brain regions in an actuate neurotoxin poisoning animal model were utilized in this work. Here, phenobarbital (PHB), which belongs to the barbiturate class of drugs, was selected as the typical representative of neurotoxins (Rubio et al., 2022), since PHB was not only abused but also often deliberately overdose for suicide or other purposes (resulting in acute and chronic intoxication) (Lindberg et al., 1992). The brain is the main target organ of PHB and it is mainly manifested as the inhibition of the central nervous system and the level changes of neurochemicals when acute poisoning occurs (Loscher and Rogawski, 2012). Up to the present, many studies have demonstrated that PHB exposure could affect the level of neurochemicals including acylcarnitines, but these studies mostly focused on the level of neurochemicals in plasma/serum (Aoki and Kuroiwa, 1982; Yanai et al., 1985; Camiña et al., 1991; Hug et al., 1991; Zelnik et al., 1995; Castro-Gago et al., 1998; Tsutsumi et al., 2020). We anticipate that the actuate poisoning animal model might clarify i) if the PHB poisoning effects on changes of plasma acylcarnitines reflect the brain acylcarnitine changes? ii) how to rationally analyze the data from LC-MS/MS for biomarker discovery.

Unlike previous works which only focused on the level of acylcarnitines in plasma or serum, in this work the levels of acylcarnitines in specific brain tissues (hippocampus, frontal lobe, striatum, and brainstem) were also detected. We believe that this work can provide an example to rationally analyze data from LC-MS/MS, profile regional differences in acylcarnitines for PHB poisoning disorders, and answer the question that “Can plasma acylcarnitines serve as toxicity biomarkers for PHB poisoning disorders?.”

## 2 Materials and methods

### 2.1 Chemicals and reagents

PHB was purchased from Shanghai Chemical Reagent Factory (Shanghai, China). Diclofenac sodium (DIC, internal standard (IS)) was obtained from Aladdin Chemical Reagent Co., Ltd. (Shanghai, China).

**FIGURE 1:**
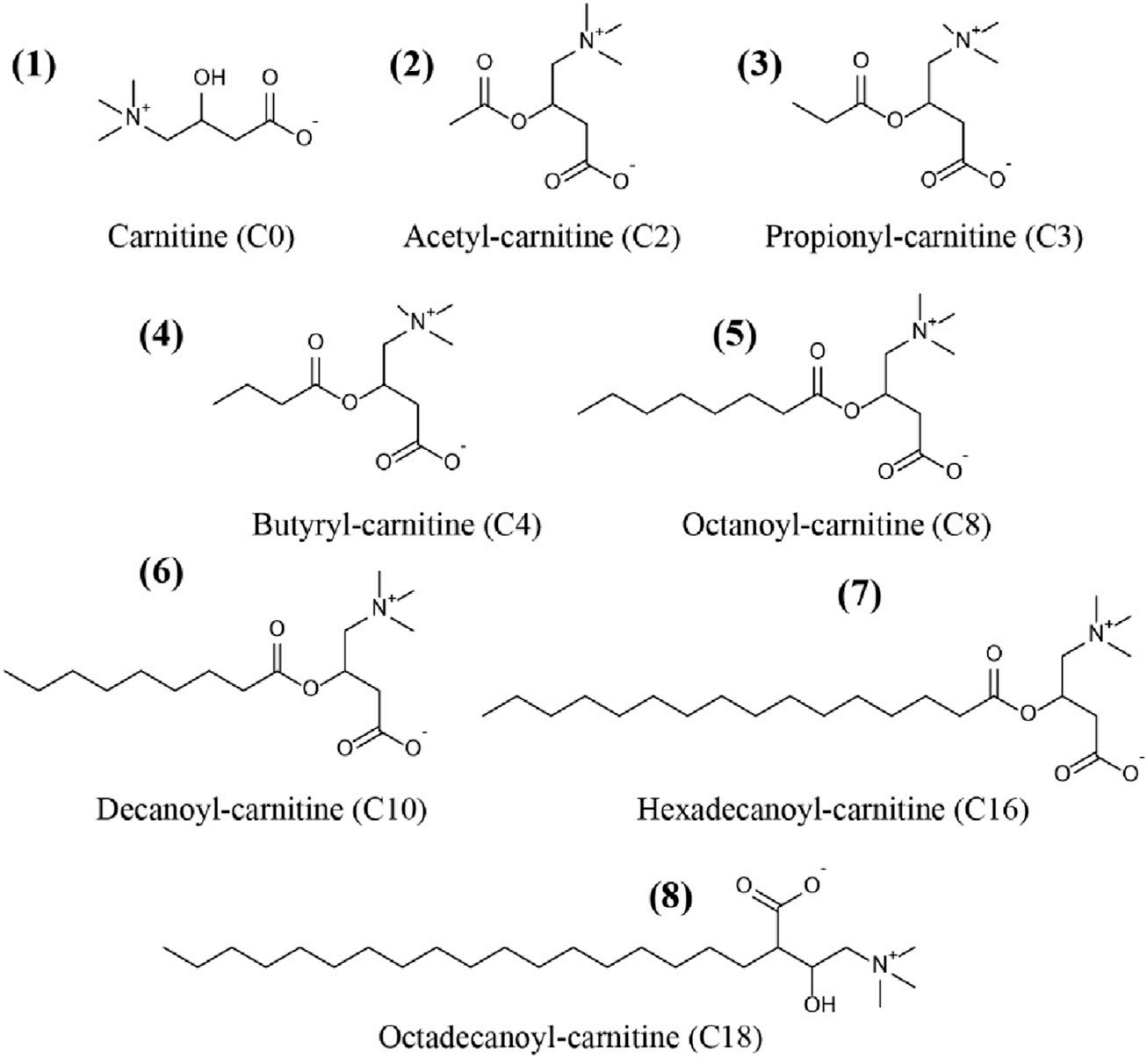
Chemical structures of the carnitine and acylcarnitines (CARs) used in this study.

Carnitine (C0), acetyl-carnitine (C2), propionyl-carnitine (C3), butyryl-carnitine (C4), octanoyl-carnitine (C8), decanoyl-carnitine (C10), hexadecanoyl-carnitine (C16), and octadecanoyl-carnitine (C18) were all purchased from TRC Corporation (Canada). Acetyl-carnitine-*d*_*3*_ (C2-*d*_*3*_), octanoyl-carnitine-*d*_*3*_ (C8-*d*_*3*_) and hexadecanoyl-carnitine-*d*_*3*_ (C16-*d*_*3*_) were supplied by Sigma Corporation (United States). Chemical structures of the CARs were showed in Figure 1.

Formic acid (FA), Methanol (MeOH), isopropanol (ISO), and acetonitrile (ACN) are of chromatographic grade, and other chemical reagents are of analytical grade. Ultrapure water was produced with a water purifier (Millipore, France).

### 2.2 Preparation of solutions and artificial plasma

The stock solutions of PHB, DIC, C0, C2, C3, C4, C8, C10, C16, C18, C2-*d*_*3*_, C8-*d*_*3*_, and C16-*d*_*3*_ were obtained by dissolving the corresponding individual analyte in methanol at 1 mg mL^-1^. The above stock solutions were stored at −20°C in the dark.

The working solution (5.0 μg mL^-1^) of PHB was prepared by diluting the stock solution of PHB with 10 mM HCl and was stored at 4°C away from light. The working solution of CARs was prepared by serially diluting the stock solution with H_2_O/ACN (1/9 = v/v) to various concentration levels. A mixture of ISs (C2-*d*_*3*_ (5 μg mL^-1^), C8-*d*_*3*_ (1 μg mL^-1^), and C16-*d*_*3*_ (1 μg mL^-1^)) was prepared by appropriate dilution of the corresponding stock solutions. All standard solutions were kept at −20°C in the dark.

The artificial plasma was prepared by dissolving 4 g of bovine albumin (BSA) in 100 mL of phosphate buffer saline solution consisting of 10 mM phosphate buffer, pH 7.4, 2.7 mM KCl, and 137 mM NaCl. The artificial plasma worked as a surrogate for the plasma matrix.

### 2.3 Animal model of acute PHB poisoning

Adult male Sprague-Dawley (SD) rats (200–250 g in weight) were purchased from The Animal Center of Tongji Medical College, Huazhong University of Science and Technology. The rats were taken and placed in the laboratory for 1 week of adaptive feeding. 31 adult male SD rats were randomly divided into five groups including four experimental groups and one control group. PHB suspension in saline was given to rats by the gavage route. The experimental groups were divided according to the LD_50_ (660 mg/kg) of oral PHB. In this study, we set oral PHB 1/8 LD_50_, 1/4 LD_50_, 1/2 LD_50_ and LD_50_ experimental groups as groups P1, P2, P3 and P4, respectively. Six rats were in P1 and P2 groups, respectively. Seven rats were in each group of P3 and P4. Five rats were in the control group. Rats in each experimental group were given 8.25 mg mL^-1^, 16.50 mg mL^-1^, 33.00 mg mL^-1^, and 66.00 mg mL^-1^ of PHB saline suspension by gavage (1 mL/100 g), respectively. Rats in the control group were given physiological saline. After 2 h of gavage (no feeding), the rats were executed by decapitation. After sacrifice, plasma and specific brain regions (In this study, specific brain regions including the hippocampus, frontal lobe, striatum, and brainstem were set as B1, B2, B3 and B4, respectively.) of each rat were collected and preserved at −80°C, respectively. The brain dissections steps were referred to the two works (Chiu et al., 2007; Spijker and Li, 2011). All animal experiments were carried out following the “Hubei Province Laboratory Animal Management Regulations”, and the experimental protocol was approved by the Laboratory Animal Ethics Committee of Huazhong University of Science and Technology ([2018] IACUC No.: 2127).

### 2.4 LC-MS/MS analysis

The detection method for PHB in plasma was referred to our previous work (Zhu et al., 2019). As for the separation of CARs from the plasma and brain tissue samples, protein precipitation (PP) was used. It is noted that before the separation, an extra homogenization step was needed. Briefly, 50 mg of rat brain tissue was homogenized after adding 100 μL of water (containing 20 mM ascorbic acid) for 2 min at 60 rpm with Freezer Mixer (Shanghai Jingxin Industrial Development Co. Ltd., China). To achieve the precipitation of protein in the biological samples, 100 μL of brain homogenates or plasma, 4 μL of internal standard solution and 500 μL of ACN containing 0.1% FA were added into a 2 mL centrifuge tube. The mixture was vortexed for 1 min followed by centrifugation at 12,000 rpm for 5 min. All the supernatant was then evaporated to dryness under nitrogen blowing, reconstituted with 100 μL of H_2_O/ACN (1/9, v/v), and centrifuged again for 5 min to collect the supernatant for LC-MS/MS analysis.

Chromatographic separations of CARs were performed on an ACQUITI UPLC BEH Amide column (1.7 μm, 2.1 × 150 mm, Waters Corporation, United States) combined with an ACQUITI UPLC BEH Amide pre-column (Waters Corporation, United States) with a column temperature of 45°C. The mobile phase consisted of solvent A (1 mM NH_4_Ac and 0.1% FA in H_2_O/ACN (v/v = 95/5)) and solvent B (1 mM NH_4_Ac and 0.1% FA in H_2_O/ACN (v/v = 5/95)). More detailed information for UHPLC-MS/MS was provided in the Supplementary Material (Supplementary Tables S1, S2).

### 2.5 Statistical analysis

Cluster analysis is an exploratory data analysis tool to sort different variables into groups so that the degree of association between two variables is maximal if they belong to the same group. As we measured the levels of CARs in the plasma and the regions of the hippocampus, frontal lobe, striatum, and brainstem, cluster analysis was performed for each group (P1, P2, P3 and P4) to determine which classes of CARs co-varied. The Variance Analysis Trend Test was used to explore the correlation between the concentrations of CARs (in plasma and different brain regions) and plasma PHB concentrations. The trend *P*-value (*P*_trend_-value) was calculated. In order to confirm this correlation, the rank correlation coefficients (*r*_s_) and *P* values of the plasma PHB and CARs in plasma and different brain regions were calculated using Spearman’s rank correlation test, and scatter plots were drawn. To investigate the correlation of plasma CARs with CARs in the brain, the *r*_s_ and *P* values of the plasma CARs and CARs in different brain regions were also calculated using Spearman’s rank correlation test, and scatter plots were drawn. The level of significance was set at 0.05 for all statistical analyses. All statistical analyses used SPSS 26 and R 3.4.1 with the R packages including “pheatmap”, “ggplot2” and “EasyDescribe”.

To screen the differential metabolites (CARs), principal component analysis (PCA) and partial least squares discriminant analysis (OPLS-DA) were conducted by using SIMCA-P 17.0 software (Umetrics AB, Umea, Sweden). To examine the modeling effect of OPLS-DA, the permutation test was conducted. Variable importance plots (VIPs) were also utilized to select putative biomarkers for PHB-induced toxicity. SPSS 26 software was used to conduct receiver operating characteristic (ROC) curve analysis illustrating the reliability of biomarkers by demonstrating both the specificity and sensitivity of the biomarker.

## 3 Results

### 3.1 Determination of CARs in plasma and brain by LC-MS/MS

In this work we developed an LC-MS/MS method for the determination of CARs in brain tissues. The developed method was also validated with respect to the limit of detection (LOD), the limit of quantification (LOQ), linear range, precision and accuracy. Detailed information about the preparation of calibration standards and quality control solutions was provided in the Supplementary Material (Supplementary Tables S3, S4).

Plasma samples were validated by the surrogate matrix method (artificial plasma), and brain tissue homogenates were validated by the background subtraction method. As seen in Supplementary Tables S3, the LOD and LOQ of CARs (C0, C2, C3, C4, C8, C10, C16, and C18) in the present LC-MS/MS detection for plasma were 0.1–1.8 ng mL^-1^ and 0.4–6.1 ng mL^-1^, respectively. In brain tissue samples, the LOD and LOQ of CARs (C0, C2, C3, C4, C8, C10, C16, and C18) were 0.1–1.0 ng mL^-1^ and 0.4–3.4 ng mL^-1^, respectively. For both plasma and brain tissue samples, the methods in the detection concentration range showed good linearity (*R* ^*2*^ > 0.99).

The precision (%) and accuracy (%) of the present method were further evaluated, and the results are listed in Supplementary Table S4. It is seen in Supplementary Tables S4 that the precision (%) and the accuracy (%) of all target analytes in plasma ranged from 0.7 to 19.2 and 80.6–119.6, respectively. In brain homogenate samples, the precision (%) and the accuracy (%) of all target analytes ranged from 0.2 to 18.9 and 80.0–118.9, respectively. Matrix effects for all analytes in brain homogenate samples ranged from 83.7% to 118.3%. The developed LC-MS/MS analytical methods were then used for determining the concentration of CARs in poisoned rats for acute PHB poisoning in P1, P2, P3, and P4. The concentrations of CARs in the plasma and specific brain regions were provided in the supplementary information (Supplementary Table S6). It is seen in Supplementary Table S6, plasma C0 concentration and brain C0 concentration are approximately the same. The short-chain acylcarnitines (C2 and C3) were generally higher than the middle-chain acylcarnitines (C8 and C10) and the long-chain acylcarnitines (C16 and C18) for most samples. Plasma C16 concentrations were generally lower than brain C16 concentrations and brain C16 concentrations were comparable to brain short-chain acylcarnitine concentrations.

### 3.2 Cluster analysis

**FIGURE 2:**
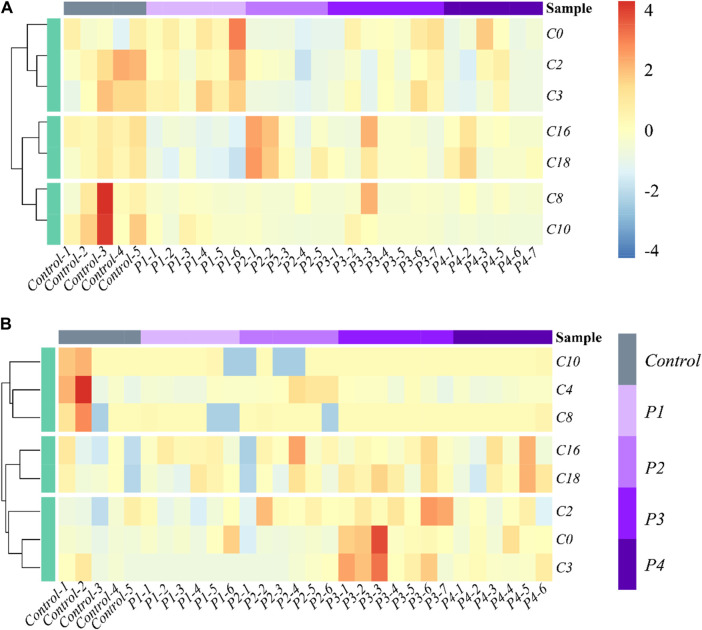
Heatmap and hierarchical clustering of **(A)** plasma and **(B)** hippocampus in the profile of CARs in acute PHB poisoning model for P1, P2, P3, and P4, compared with controls. Control-1 to 5 and P1-1 to P4-7 are the sample numbers of the control group and experimental groups, respectively.

The effects of PHB intoxication on CARs in different specific brain regions have not been investigated in previous works. Therefore, there is currently no information on the potential changes in the spectrum of CARs induced by acute PHB poising in different specific brain regions in the literature. To reflect the metabolite (CARs) content at different concentrations of PHB intoxication and observe metabolite clustering, in this work a hierarchical clustering analysis was performed based on the metabolite content and the results were represented as a heat map (Figure 2; Supplementary Figure S4). This cluster analysis generally revealed that, in the plasma and the brain regions except for the frontal lobe, three clusters have the same metabolite pattern including C0 and short-chain acylcarnitine (C2, C3, and C4), medium-chain acylcarnitine (C8 and C10) and long chain acylcarnitine (C16 and C18) clusters (see Figures 2A, B; Supplementary Figure S4B, C). For the frontal lobe, C2, C4, C16, and C18 changed into the same metabolism pattern (Supplementary Figure S4A). Overall, the levels of most CAR species decreased upon PHB poisoning in plasma, and some CAR species increased in the brain. These results illustrate that PHB poisoning may result in different CAR metabolism in plasma and brain tissues.

### 3.3 Plasma CARs inadequately reflect brain CARs metabolism

#### 3.3.1 Correlation between PHB and CARs in plasma and brain

**FIGURE 3:**
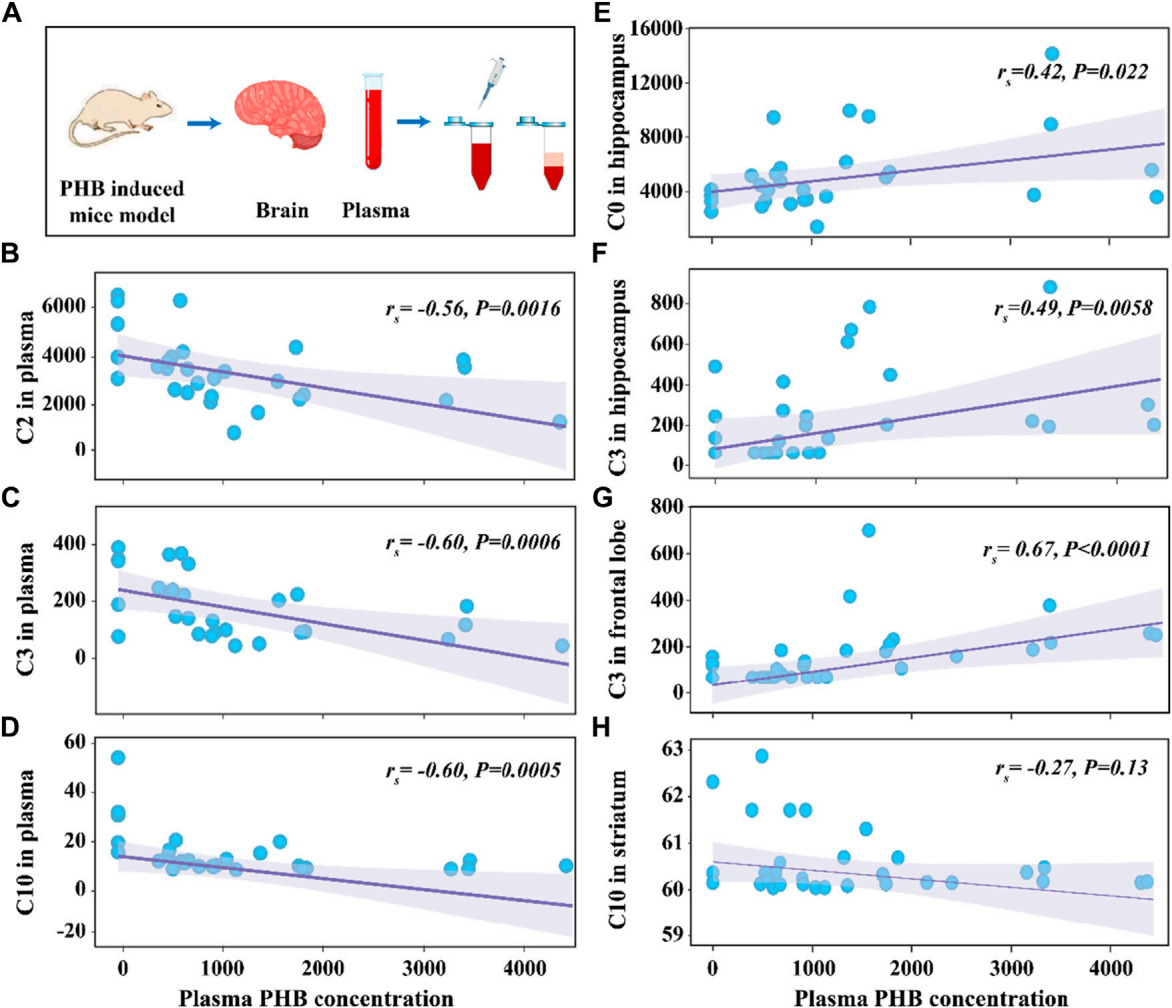
Schematic illustration of the experimental process **(A)**. Scattering plot for the correlation of C2 in plasma **(B)**, C3 in plasma **(C)**, C10 in plasma **(D)**, C0 in hippocampus **(E)**, C3 in hippocampus **(F)**, C3 in frontal lobe **(G)**, and C10 in striatum **(H)** with plasma PHB concentration. Note: “*r*_s_” indicates Spearman correlation coefficient; *p* < 0.05 means a linear correlation; The y-axis is the levels of acylcarnitines (ng mL^-1^) in corresponding samples and the x-axis is the plasma PHB concentration (μg mL^-1^).

The average concentrations of plasma PHB were 507 μg mL^-1^, 942 μg mL^-1^, 1,358 μg mL^-1^ and 2292 μg mL^-1^ in the P1, P2, P3, and P4 group, respectively (Supplementary Table S5). To explore the correlation between the concentrations of CARs and PHB in plasma and brain, the Variance analysis trend test was used to calculate the *P*_trend_-value. The groups were divided according to the quartiles of PHB, and the CAR concentrations in each group were expressed as median and interquartile spacing (see Supplementary Table S7). Supplementary Table S7 shows the associations between concentrations of plasma PHB and CARs in plasma and brain. It is seen that the *P*_trend_-values for C2, C3, and C10 were all less than 0.05 (C2-*P*_trend_ = 0.003, C3-*P*_trend_ = 0.001, and C10-*P*_trend_ = 0.002), indicating the plasma PHB concentrations in the 2nd, 3rd and 4th quartiles had lower plasma C2, C3, and C10 concentrations than that in the 1st quartile. To confirm these results, the Spearman rank correlation test was supplemented to calculate *r*_s_ and *P*-values (see Figure 3). The negative associations between plasma PHB and C2, C3, and C10 were also shown in Figure 3. C2 (*r*_s_ = −0.56, *p* = 0.0016; Figure 3B), C3 (*r*_s_ = −0.60, *p* = 0.0006; Figure 3C), and C10 (*r*_s_ = −0.60, *p* = 0.0005; Figure 3D) level in plasma were negatively correlated with the plasma PHB concentrations. In other words, dose-dependent decrement was observed in the concentrations of plasma C2, C3 and C10.

To present the statistically significant results in Supplementary Table S7 more visually, the bar charts were plotted in Supplementary Figure S5. As seen in Supplementary Figure S5, Plasma C2 (*P*_trend_ = 0.003), C3 (*P*_trend_ = 0.001), and C10 (*P*_trend_ = 0.002) show a decreasing trend with increasing PHB concentrations. Besides, compared with the 1st plasma PHB quartile, the levels of plasma C2, C3, and C10 in the high-dose PHB groups (3rd/4th quartile) were significantly decreased (*P* < 0.05). Conversely, plasma C0, C8, C16, and C18 were not significantly associated with PHB exposure (Supplementary Table S7; Supplementary Figure S5).

The relationship between plasma PHB and broad-spectrum CAR changes in specific brain regions remained unclear. In order to clarify this, correlation between PHB and acylcarnitines in brain was also studied using the similar way. It is seen that, for the brainstem, none of the tested CARs was significantly associated with PHB exposure (Supplementary Table S7; Supplementary Figure S9). However, for the other three brain regions, some CARs showed significantly associated with PHB exposure: i) C0 and C3 levels in hippocampus were positively correlated with the plasma PHB concentrations (Supplementary Figure S6A, C); ii) C3 level in frontal lobe was positively correlated with the plasma PHB concentrations (Supplementary Figure S7B); and iii) C10 level in striatum was weak negatively correlated with the plasma PHB concentrations (see Figure 3H; Supplementary Figure S8F). To confirm these results, the Spearman rank correlation test was supplemented to calculate *r*_s_ and *P*-values (see Figure 3). The positive associations between plasma PHB and hippocampus-C2, hippocampus-C3, and frontal lobe-C3 were also shown in Figure 3. Hippocampus-C2 (*r*_s_ = 0.42, *p* = 0.022; Figure 3E), hippocampus-C3 (*r*_s_ = 0.49, *p* = 0.0058; Figure 3F), and frontal lobe-C3 (*r*_s_ = 0.67, *p* < 0.0001; Figure 3G) level in plasma were positively correlated with the plasma PHB concentrations. However, the C10-*r*_s_ = −0.27, *p* = 0.13 (Figure 3H) in striatum showed no linear relation, so the result should be interpreted with caution. In other words, dose-dependent increment was observed in the concentrations of hippocampus-C2, hippocampus-C3 and frontal lobe-C3.

#### 3.3.2 Correlation between CARs in plasma and CARs in brain

To investigate whether plasma CAR levels corresponded to CAR levels in brain tissues, the Spearman rank correlation test was further used to calculate *r*_s_ and *P*-values for this correlation (Supplementary Figure S10). It is seen that no significant correlation between plasma CARs (C0 and C10) and their counterparts in any brain region was found. For other plasma CARs, weak correlations (0.3 < |*r*_s_| < 0.5) (Altman, 1991) between them and their brain counterparts could be found (Fig. S10), including between plasma C2 and B3 C2 (*r*_s_ = −0.409, *p* = 0.027), plasma C3 and B4 C3 (*r*_s_ = −0.368, *p* = 0.049), plasma C3 and B2 C3 (*r*_s_ = −0.399, *p* = 0.032), plasma C8 and B2 C8 (*r*_s_ = −0.402, *p* = 0.031), plasma C16 and B2 C16 (*r*_s_ = −0.420, *p* = 0.023) and plasma C18 and B2 C18 (*r*_s_ = −0.383, *p* = 0.040).

### 3.4 Biomarker screening using two different strategies

In the field of biomarker discovery, using a selected dose group and a combined group were the most commonly used strategies to screen potential biomarkers (Motsinger-Reif et al., 2013; Ezaki et al., 2017; Varma et al., 2018). In this study, the combination of groups was established based on the results of the hot plate test (Supplementary Table S8; Supplementary Figure S11). It is seen that the hot plate reaction times of mice in groups P2, P3 and P4 after gavage of PHB reached 60 s, indicating the degree of intoxication in groups P2, P3 and P4 was approximately the same. However, the hot plate reaction times of mice in group P1 were in the range of 4–20 s, which was different from those in group P2, P3 and P4 after gavage of PHB (*p* < 0.05), indicating the degree of intoxication in group P1 was different from that in group P2, P3 and P4. Therefore, only the first strategy was used to screen for biomarkers in the P1 group. While for the P2, P3 and P4 groups, both the first strategy and the second strategy were utilized to screen the biomarkers.

#### 3.4.1 Screening potential biomarkers using the first strategy

In metabolomics research, the general steps of biomarker identification include four steps: i) establishment of the metabolic profile; ii) confirmation of specific metabolites; iii) inferring the biological metabolic pathway; iv) validation of biomarkers (Liao et al., 2012). Confirmation of specific metabolites is one of the most important steps in screening the biomarker, where various statistical analysis methods were used. Univariate analysis method, including parametric test and non-parametric test, is usually used in metabolomics research to quickly investigate the differences of metabolites in different categories (Saccenti et al., 2014). However, univariate analysis cannot reveal the complex interaction among variables because of the high dimension of metabolomics data, therefore multivariate statistical analysis including PCA and OPLS-DA was used in the present study.

**FIGURE 4:**
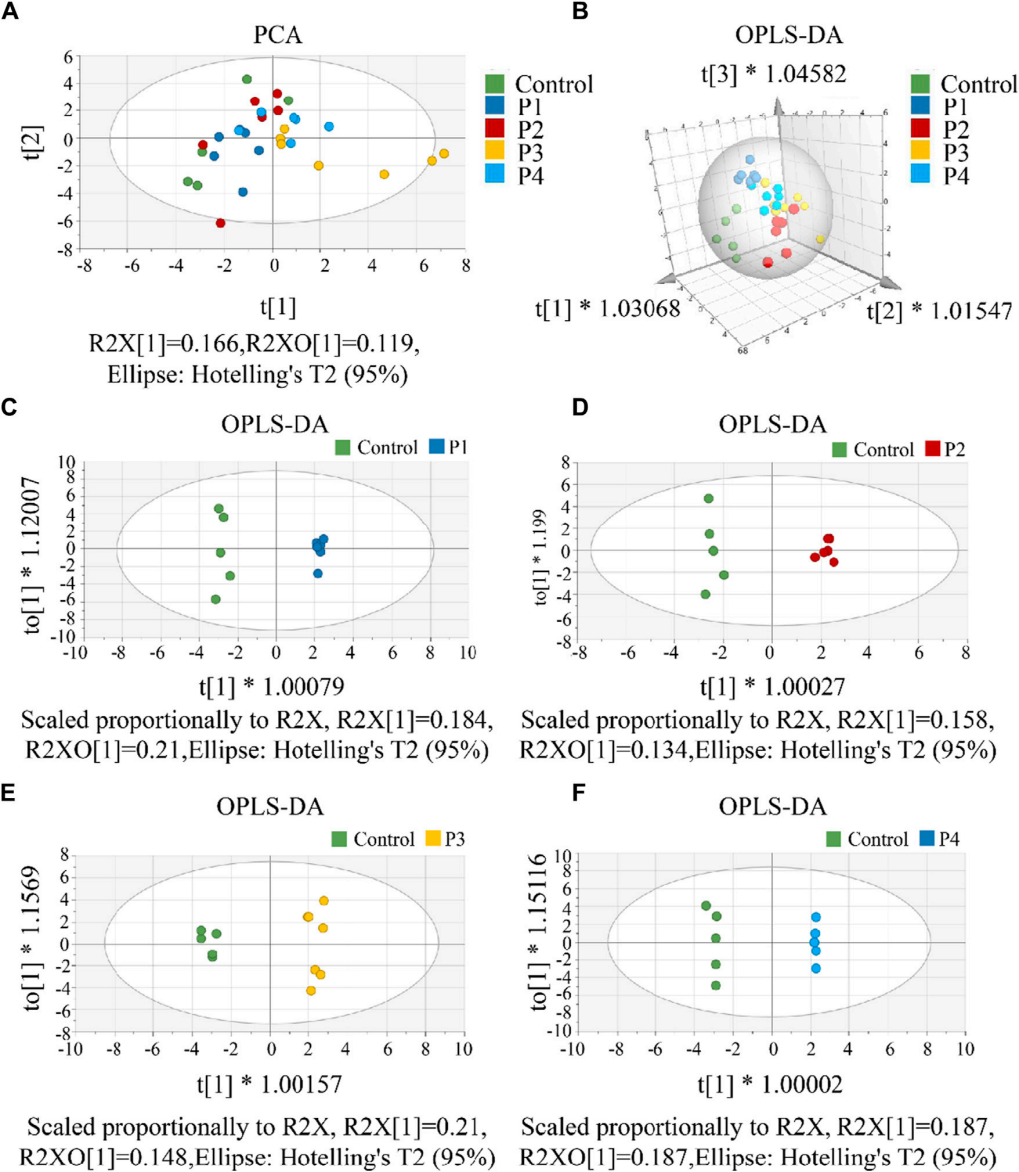
**(A)** The PCA score scatter plot that shows plasma and brain metabolic differences. The x-axis of the PCA score plot represents the score value of each sample projected on the first principal component (PC1) and the y-axis represents the score value of each sample projected on the second principal component (PC2). **(B)** The OPLS-DA spatial score scatter plot that shows plasma and brain metabolic differences. OPLS-DA score scatter plot of **(C)** P1, **(D)** P2, **(E)** P3 and **(F)** P4 compared with control groups, respectively. The x-axis of the OPLS-DA score plot indicates the score values of the principal components, which reveal differences between groups. The y-axis indicates the score values of the orthogonal components, which reveal differences within groups (differences between samples within groups).

First, a single dose group (P1, P2, P3, or P4) was separately selected to compare with the control group to screen differential metabolites. To investigate CAR changes in plasma and brain, the control, P1, P2, P3, and P4 groups were investigated using PCA analysis. As shown in Figure 4A, all these groups were not well distinguished in terms of PCA score plots. Compared to unsupervised PCA, supervised OPLS-DA was able to get the variables that caused differences among groups. Therefore, OPLS-DA was applied (Figures 4B–F) for these five groups, and the main parameters of the model were listed in Supplementary Table S9. As anticipated, the separation of the five groups was much better than that in the PCA analysis. Usually, three indicators (R2X, R2Y, and Q2Y) are used to evaluate the fitting effect of the OPLS-DA model. R2 describes how well the model is fitted. Q2 describes how well the model is predicted. The values of R2 and Q2 range from 0 to 1, where one indicates perfect fitness and predictivity (Huang et al., 2018). As seen in Supplementary Table S9, the values of R2X, R2Y, and Q2Y were in the range of 0.49–0.70, 0.98–0.99, and 0.70–0.79, respectively. These values of three indicators in the OPLS-DA models were acceptable. Moreover, a permutation test (Supplementary Figure S12) confirmed this OPLS-DA model was not over-fitted. All these data analyses indicated the existence of one or more variables that caused differences among groups.

**FIGURE 5:**
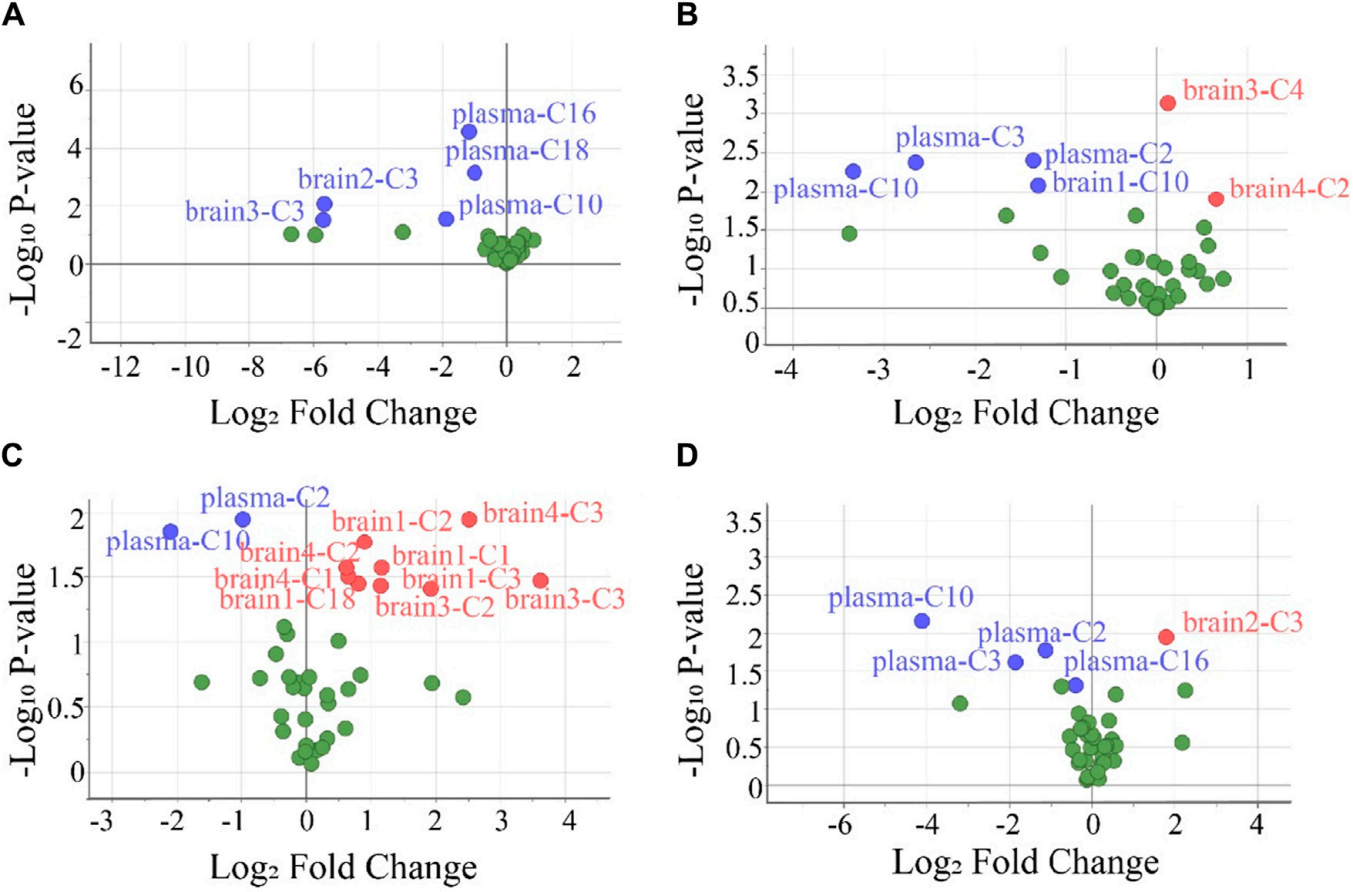
Volcano plots were used to select the candidate biomarkers. CARs exhibited statistically significant changes after the administration of **(A)** P1, **(B)** P2, **(C)** P3, and **(D)** P4, respectively. The x-axis represents the multiplication relationship after logarithmic transformation, and the y-axis represents the *p*-value after logarithmic transformation. The dots marked with blue indicate that the CARs significantly downregulated, the dots marked with red indicate that the CARs significantly upregulated and the green dots represent no significant CARs.

To screen the differential variables (potential biomarkers), the values of VIP and -Log_10_ *P* in OPLS-DA were analyzed in detail (Supplementary Table S10). Generally, VIP >1 and -Log_10_ *p*-value >1.3 (*p-*value <0.05 in the Student’s t-test) in OPLS-DA indicate a potential biomarker (Kuhl et al., 2012). Using this criterion, biomarkers in the plasma and all brain tissues could be found. To visually show the potential biomarkers in the different biological samples, volcano maps were presented by reuse of the selected data in the OPLS-DA model (Figure 5). Similarly to that in Supplementary Table S10, the volcano map showed different potential biomarkers (blue dots and red dots) in the four groups. Moreover, each group possessed upregulated biomarkers (red dots with a Log_2_ Fold Change >0) and downregulated biomarkers (blue dots with a Log_2_ Fold Change <0).

**FIGURE 6:**
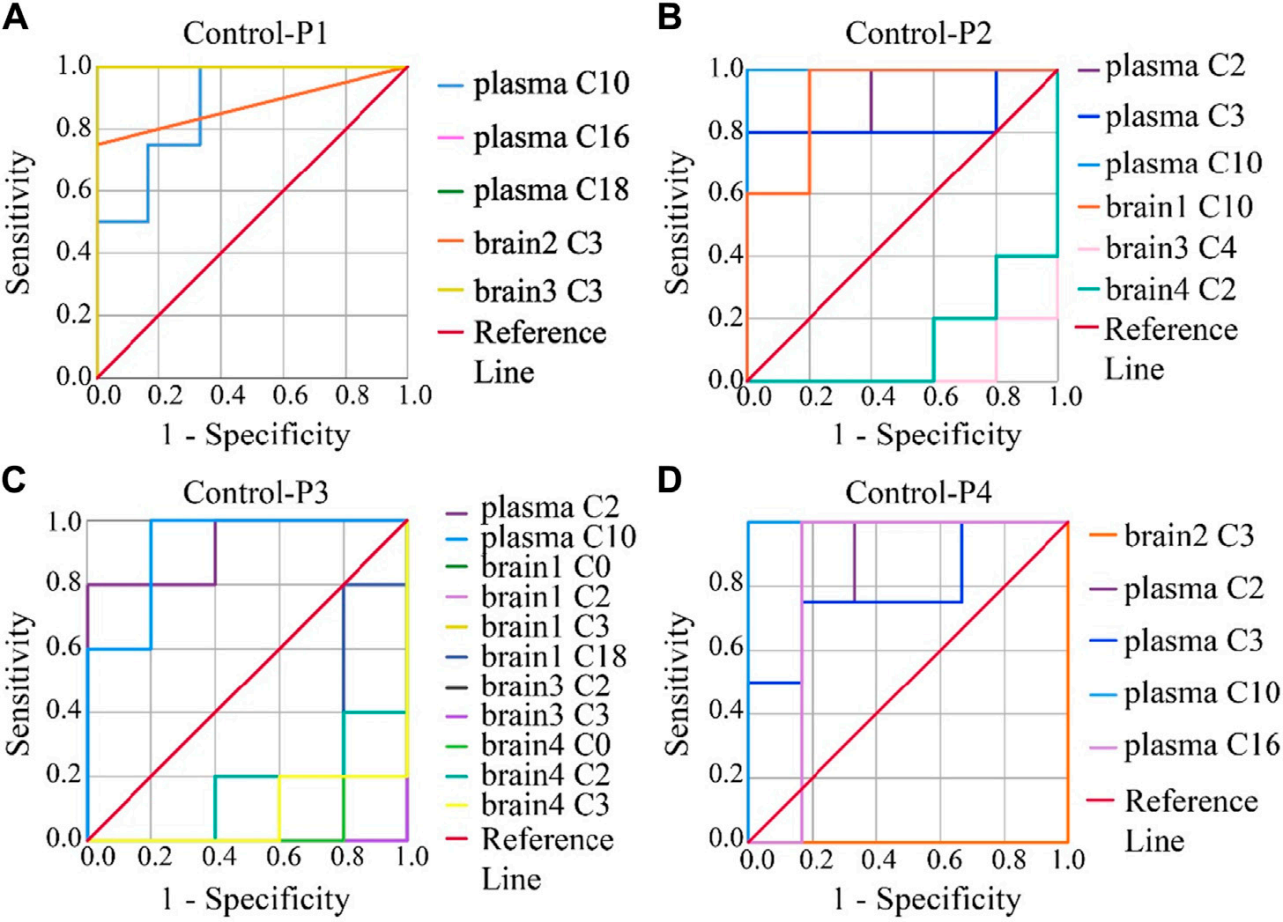
ROC analysis was used to show the sensitivity and specificity of the CAR data. Biomarkers reconfirm by ROC analysis of the plasma and brain CAR data. The reconfirmation of the potential biomarkers exhibited statistically significant changes after the administration of **(A)** P1, **(B)** P2, **(C)** P3, and **(D)** P4, respectively. The smaller the x-axis is, the higher the accuracy will be. The larger the y-axis is, the better the accuracy is. The AUC represents the accuracy of prediction. AUC >0.8 indicated good predictive ability.

To evaluate the accuracy of potential variables (in the volcano map), ROC diagnostic analysis was investigated by examining the area under curves (AUCs) of these biomarkers (Figure 6). Generally, if the AUC >0.8, the biomarkers can be employed as good biomarkers (Mandrekar, 2010; Martin et al., 2022). Accordingly, if the AUC of biomarkers was greater than 0.8 in our work, that can be considered to play an important role in PHB-induced toxicity. As shown in Figure 6 and Supplementary Table S12 (detailed AUC values of potential biomarkers), 5, 4, 2, and 3 metabolites were obtained as important biomarkers in P1, P2, P3, and P4 groups, respectively. From these 14 metabolites, a potential toxicity biomarker group was obtained including eight downregulated CARs (plasma C2, plasma C3, plasma C10, plasma C16, plasma C18, brain1 C10, brain2 C3, brain3 C3) with AUC >0.8.

#### 3.4.2 Screening potential biomarkers using the second strategy

**FIGURE 7:**
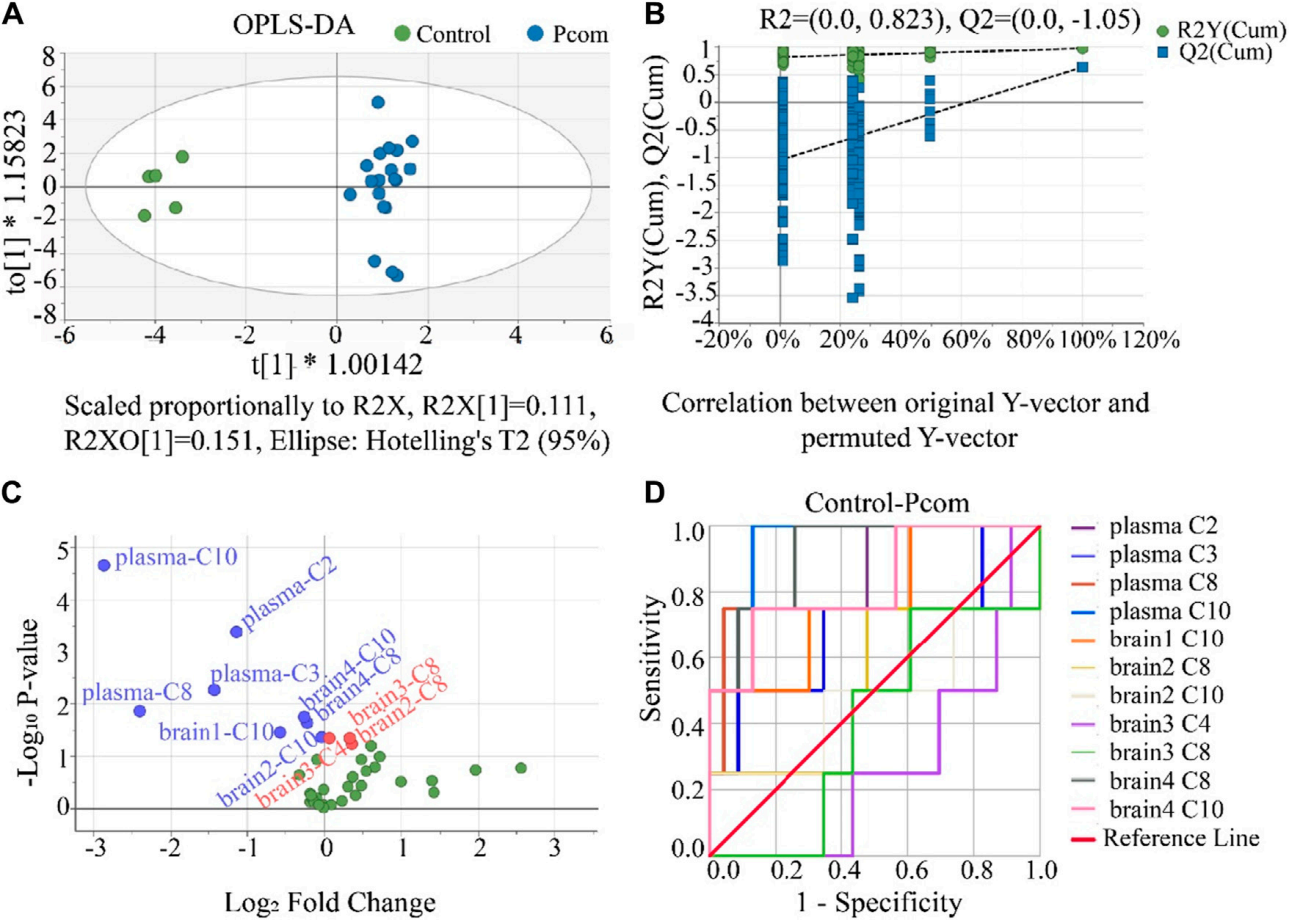
**(A)** Result of the OPLS-DA score scatter plots that show plasma and brain metabolic differences between the Pcom and the control group. **(B)** Result of the permutation test, the modeling effect of OPLS-DA of Pcom compared with the control group. *Y*-axis intercepts: R2 = (0.0, 0.823) and Q2 = (0.0, −1.05). **(C)** Volcano plot analysis of the plasma and brain CAR data. The x-axis represents the multiplication relationship after logarithmic transformation, and the y-axis represents the *p*-value after logarithmic transformation. The green dots represent no significant metabolites. **(D)** ROC analysis was used to show the sensitivity and specificity of the CAR data.

Comparing the control group with the one combined poisoning group was another strategy for potential biomarker screening (Motsinger-Reif et al., 2013; Varma et al., 2018). Here, this method was also investigated. According to the hot plate reaction time after gavage, the concentration groups of P2, P3, and P4 were combined into one combined poisoning group (Pcom). First, the OPLS-DA model was applied to the Pcom group (Figure 7A). Unlike that in Figure 4A (using a selected dose group), the separation of the two groups was significant (Figure 7A). The values of the three indicators R2X, R2Y and Q2Y in the OPLS-DA model were 0.5, 0.9 and 0.6 (Supplementary Table S9) respectively, suggesting that the OPLS-DA models were acceptable.

Our permutation test results show that the crossing point of the regression line of Q2 on the y-ordinate was less than 0 (Figure 7B), indicating the above OPLS-DA model was not over-fitted. Using the same criterion, biomarkers in the plasma and all brain tissues could be found (Supplementary Table S11). Using these data, the volcano plot analysis was further drawn to visualize the results of screening differential metabolites (Figure 7C). It is seen in this figure that 11 metabolites were obtained as important biomarkers, including eight downregulated biomarkers (blue dots with a Log_2_ Fold Change <0) and 3 upregulated biomarkers (red dots with a Log_2_ Fold Change >0). These 11 potential variables were further evaluated using ROC diagnostic analysis (Figure 7D). As shown in Figure 7D; Supplementary Table S12 (detailed AUC values of potential biomarkers), a potential toxicity biomarker group was obtained, containing five downregulated CARs (plasma C2, plasma C8, plasma C10, brain4 C8, brain4 C10) with AUC >0.8.

## 4 Discussion

Previous works have successfully reported the determination of CARs in plasm by LC-MS/MS analysis (Giesbertz et al., 2015; Minkler et al., 2015; Scott et al., 2016). However, there are few reports on the determination of CARs in brain tissue by LC-MS/MS. Compared to the determination of CARs in plasm, simultaneous measurement of the CARs in brain tissues is a much more challenging task, mainly due to difficulties caused by the lower abundance of CARs, diversity of their structural and physicochemical properties, more complex matrix interference, and potential instability. Moreover, during the neurotoxicology studies (to reveal the effects of plasma neurotoxin on the neurochemical levels in specific brain regions), it is highly demanded a simple, reliable and comprehensive analytical method for CARs in brain tissue to obtain an accurate investigation of the dosage effect in the acute neurotoxin model (e.g., PHB). To meet these requirements, in this work we developed an LC-MS/MS method for the determination of CARs in brain tissues.

According to the literature, the normal total plasma C0 and acylcarnitine concentrations have generally been reported as 45–60 μmol L^-1^ and 6–9 μmol L^-1^, respectively. The concentration of total C0 and acylcarnitines in the brain of an adult rat is about 400 nm g^-1^ and 380 nm g^-1^, respectively (Morris and Carey, 1983; Nakano et al., 1989; Reuter et al., 2008; Reuter and Evans, 2012). These values were among the linear range of the present LC-MS/MS detection. This indicates the LC-MS/MS method is suitable for the determination of CARs in plasma and brain tissues. All validation results of precision (%) and accuracy (%) further support that the method based on PP-LC-MS/MS is accurate and reliable for the quantification of CARs in plasma and brain.

The plasma C0 concentration increased from the 1st to the 2nd quartile and then decreased in the 3rd and 4th quartile, which were similar to those previous studies (Camiña et al., 1991; Hug et al., 1991). As for plasma C2, C3 and C10, their concentrations were negatively correlated with PHB plasma concentrations. This negative correlation might be due to the decrease in renal reabsorption of free C0 (Matsuda et al., 1986; Matsuda and Ohtani, 1986; Rodriguez-Segade et al., 1989; Camiña et al., 1991) and the increase in urinary excretion of acylcarnitines caused by intoxication (Morris and Carey, 1983; Millington et al., 1985). Many researches have already demonstrated that barbiturates exposure could affect the level of serum CARs, but CAR levels were primarily measured in serum instead of specific brain regions (Camiña et al., 1991; Hug et al., 1991; Zelnik et al., 1995; Castro-Gago et al., 1998). From the perspective of the results in this work that reached statistical significance (*P*_trend_ < 0.05), in contrast to the decrease of plasma C2, C3, and C10 with the increase of PHB, an increase of C0 and C3 in hippocampus and an increase of C3 in frontal lobe were found. Although the changes in plasma CARs and brain CARs caused by PHB poisoning are different, weak negative correlations could be established between plasma CARs and their brain counterparts. However, the correlation is so weak that the changes in plasma CARs may not fully represent changes in brain CARs (Camiña et al., 1991; Schooneman et al., 2014; Dave et al., 2022). These findings suggested that PHB poisoning affects plasma and brain acylcarnitines differently, and the changes in plasma acylcarnitines may not fully represent changes in brain acylcarnitine.

Many previous works involved in biomarkers screening, the screening process was finished at the above steps (Motsinger-Reif et al., 2013; Ezaki et al., 2017; Varma et al., 2018). Under the first strategy, 14 potential toxicity biomarkers were obtained including eight downregulated CARs with AUC >0.8. Under the second strategy, 11 potential toxicity biomarkers were obtained containing five downregulated CARs with AUC >0.8. However, it is seen in the above analysis that, the biomarkers in plasma screened by both strategies were different, especially for the biomarkers in the brain. Principles of biomarker screening included that the repeatability of biomarkers for a poison exposure should be within acceptable limits and the selected biomarkers must have certain specificity were considered in this work.

As an important principle, a biomarker should also possess a good dose-response relationship (McNamara et al., 2020; Pang et al., 2021). However, the above results did not consider the dose-response relationship between PHB level in plasma and screened biomarker level in plasma and brain. In this case, we considered that the screening biomarkers using the above two strategies were not enough. Thus, the dose-response relationship should be considered in biomarker screening. Under the first strategy, the result of screened biomarker (C10) in the P1 group is consistent with the dose-response relationship (the concentration of plasma C10 decreased with the increase of PHB concentration, see Figure 3D), and the result of screened biomarkers (C2 and C10) in P2, P3 and P4 groups were consistent with the dose-response relationship (Figures 3C, D). Under the second strategy, the result of screened biomarkers (downregulated plasma C2, and C10) are consistent with the dose-response relationship (Figures 3C, D). Obviously, after considering the dose-response relationship, the results of the biomarker screening were altered, and the types of biomarkers using the first strategy and the second strategy were consistent. It is noted that there were no biomarkers in the brain under both two strategies after considering the dose-response relationship. These results also show that plasma acylcarnitines could serve as toxicity biomarkers for PHB poisoning disorders, while no biomarkers in the brain were found. Although no biomarkers of PHB poisoning were found in the brain, PHB affected the level of CARs in the brain.

## 5 Conclusion

In this study, we presented an example to rationally analyze data from LC-MS/MS. With this study, the differences of CARs in plasma and brain regions for PHB poisoning disorders were profiled and the question that “Can plasma acylcarnitines serve as toxicity biomarkers for PHB poisoning disorders?” was answered. Based on the PHB poisoning model, this work considered biomarker screening principles including certain specificity, repeatability, and dose-response relationship to rationally analyze data from LC-MS/MS for biomarker discovery. We found that plasma C2 and C10 might serve as toxicity biomarkers for PHB poisoning disorders. In addition, very weak correlations between CARs in plasma and counterparts in specific brain regions were found, suggesting changes in plasma CARs may not be fully representative of changes in brain CARs. In short, our work profiled CAR changes in plasma and brain tissues and provided theoretical support for further research on the neurotoxic mechanism.

However, there were some shortcomings in this study: i) Only a representative portion of CARs was selected, which may have missed information on other CARs; ii) the number of mice in each group was not large enough, which may make some statistical error; iii) other tissues such as liver, heart, muscle, and fat tissues were not collected for CAR analysis to describe the acylcarnitine turnover in the mice, and the acylcarnitine efflux or release from the brain either (Dambrova et al., 2022); iv) samples of multiple periods were not collected to observe the changes in CARs over time.

In summary, to obtain detailed information about the biomarkers of different exposure levels, it is recommended to screen biomarkers by the first strategy. To obtain general information about the biomarkers of overall exposure, it is recommended to screen biomarkers by the second strategy. For the biomarker screening analysis of LC-MS/MS data, since dose-response relationships are one of the principles of biomarker selection, the dose-response relationship should be considered for both the first and second biomarker screening strategies. After the biomarker screening, it is necessary to further complete the verification of biomarkers in a larger population. Only rational analysis can find more accurate biomarkers and provide a solid foundation for further verification of biomarkers.

## Data Availability

The original contributions presented in the study are included in the article/Supplementary Material, further inquiries can be directed to the corresponding authors.

## Abbreviations

PHB Phenobarbital; CARs Carnitine and acylcarnitines; C0 Carnitine; C2 Acetyl-carnitine; C3 Propionyl-carnitine; C4 Butyryl-carnitine; C8 Octanoyl-carnitine; C10 Decanoyl-carnitine; C16 Hexadecanoyl-carnitine; C18 Octadecanoyl-carnitine; FA, Formic acid; MeOH Methanol; ISO Isopropanol; ACN Acetonitrile; SD Sprague-Dawley; PP Protein precipitation; LOD Limit of detection; LOQ Limit of quantification; PCA Principal component analysis; OPLS-DA Partial least squares discriminant analysis; VIPs Variable importance plots; ROC Receiver operating characteristic; PC1 First principal component; PC2 Second principal component.
