# Supplementary material for: Rational analysis of data from LC-MS/MS: new insights in acylcarnitines as biomarkers for brain disorders or neurotoxicity
Source: Front Pharmacol. 2024 Aug 22;15:1441755. doi: 10.3389/fphar.2024.1441755 (PMC11374737; doi:10.3389/fphar.2024.1441755)
Supplement: Supplementary file 1 [file DataSheet1.docx]

**Supporting Information**

**Rational analysis of data from LC-MS/MS: New insights in acylcarnitines as biomarkers for brain disorders or neurotoxicity**

Li Chen^1^, Ruiqin Zhu^2^, Yaxing Ma^1^, Chuixiu Huang^2,^ *, Xiantao Shen^1,^ *

*^1^ Key Laboratory of Environment and Health, Ministry of Education & Ministry of Environmental Protection and State Key Laboratory of Environmental Health (Incubation), School of Public Health, Tongji Medical College, Huazhong University of Science and Technology, 13 Hangkong Road, Wuhan, 430030, China*

*^2^ Department of Forensic Medicine, Huazhong University of Science and Technology, 13 Hangkong Road, Wuhan, 430030, China*

** Corresponding authors:* *xtshenlab@hust.edu.cn (X. Shen)*; [chuixiuh@hust.edu.cn](mailto:chuixiuh@hust.edu.cn) *(C. Huang)*

**Figure S1.** Chemical structures of the carnitine and acylcarnitines used in this study.

**Figure S2.** Recovery of protein precipitation extraction of **(A)** C0, C2, C3 and C4, **(B)** C8, C10, C16 and C18 from artificial plasma with different organic solvents.

**Figure S3.** Recovery of protein precipitation extraction of **(A)** C0, C2, C3 and C4, **(B)** C8, C10, C16 and C18 from brain tissue with different organic solvents.

**Figure S4.** Heatmap and hierarchical clustering of **(A)** plasma, **(B)** hippocampus, **(C)** frontal lobe **(D)** striatum and **(E)** brainstem in the profile of acylcarnitines in acute PHB poisoning model for P1, P2, P3, and P4, compared with controls.

**Figure S5.** Levels of **(A)** C0, **(B)** C2, **(C)** C3, **(D)** C8, **(E)** C10, **(F)** C16 and **(G)** C18 (ng mL^-1^) in plasma by quartiles of plasma PHB concentration. Range of PHB quartiles [μg mL^-1^]: 1st, < 492.09; 2nd, 492.09-772.19; 3rd, 772.20-1534.09; 4th, ≥ 1534.10. Bars correspond to estimated mean values of acylcarnitines. Whiskers indicate standard error. *P*_trend_, *P*-value for linear trend across quartiles of PHB. The acylcarnitines that showed significantly decreasing trend with increasing PHB concentrations. were highlighted with red color.

**Figure S6.** Levels of **(A)** C0, **(B)** C2, **(C)** C3, **(D)** C4, **(E)** C8, **(F)** C10, **(G)** C16 and **(H)** C18 (ng g^-1^) in hippocampus by quartiles of plasma PHB concentration. Range of PHB quartiles [μg mL^-1^]: 1st, < 492.09; 2nd, 492.09-772.19; 3rd, 772.20-1534.09; 4th, ≥ 1534.10. Bars correspond to estimated mean values of acylcarnitines. Whiskers indicate standard error. *P*_trend_, *P*-value for linear trend across quartiles of PHB. The acylcarnitines that showed significantly increasing trend with increasing PHB concentrations were highlighted with red color.

**Figure S7.** Levels of **(A)** C0, **(B)** C2, **(C)** C3, **(D)** C4, **(E)** C8, **(F)** C10, **(G)** C16 and **(H)** C18 (ng g^-1^) in frontal lobe by quartiles of plasma PHB concentration. Range of PHB quartiles [μg mL^-1^]: 1st, < 492.09; 2nd, 492.09-772.19; 3rd, 772.20-1534.09; 4th, ≥ 1534.10. Bars correspond to estimated mean values of acylcarnitines. Whiskers indicate standard error. *P*_trend_, *P*-value for linear trend across quartiles of PHB. The acylcarnitines that showed significantly increasing trend with increasing PHB concentrations were highlighted with red color.

**Figure S8.** Levels of **(A)** C0, **(B)** C2, **(C)** C3, **(D)** C4, **(E)** C8, **(F)** C10, **(G)** C16 and **(H)** C18 (ng g^-1^) in striatum by quartiles of plasma PHB concentration. Range of PHB quartiles [μg mL^-1^]: 1st, < 492.09; 2nd, 492.09-772.19; 3rd, 772.20-1534.09; 4th, ≥ 1534.10. Bars correspond to estimated mean values of acylcarnitines. Whiskers indicate standard error. *P*_trend_, *P*-value for linear trend across quartiles of PHB. The acylcarnitines that showed significantly decreasing trend with increasing PHB concentrations were highlighted with red color.

**Figure S9.** Levels of **(A)** C0, **(B)** C2, **(C)** C3, **(D)** C4, **(E)** C8, **(F)** C10, **(G)** C16 and **(H)** C18 (ng g^-1^) in brainstem by quartiles of plasma PHB concentration. Range of PHB quartiles [μg mL^-1^]: 1st, < 492.09; 2nd, 492.09-772.19; 3rd, 772.20-1534.09; 4th, ≥ 1534.10. Bars correspond to estimated mean values of acylcarnitines. Whiskers indicate standard error. *P*_trend_, *P*-value for linear trend across quartiles of PHB. No acylcarnitines showed significantly decreasing trend with increasing PHB concentrations in brainstem.

**Figure S10.** Scattering plot for the correlations of **(A)** C3, **(B)** C8, **(C)** C16, **(D)** C18, **(E)** C2 and **(F)** C3 between plasma and specific brain regions. “*r*_s_” indicates Spearman correlation coefficient. Correlation is significant at *p* < 0.05 (2-tailed). Note that the y-axis is the levels of acylcarnitines (ng g^-1^) in the specific brain region samples and the x-axis is the plasma acylcarnitine concentration (ng mL^-1^).

**Figure S11. (A)** The PCA score scatter plot that shows plasma and brain metabolic differences. The x-axis of the PCA score plot represents the score value of each sample projected on the first principal component (PC1) and the y-axis represents the score value of each sample projected on the second principal component (PC2). **(B)** The OPLS-DA spatial score scatter plot that shows plasma and brain metabolic differences. OPLS-DA score scatter plot of **(C)** P1, **(D)** P2, **(E)** P3 and **(F)** P4 compared with control groups, respectively. The x-axis of the OPLS-DA score plot indicates the score values of the principal components, which reveal differences between groups. The y-axis indicates the score values of the orthogonal components, which reveal differences within groups (differences between samples within groups).

**Figure S12** Result of the permutation test, the modeling effect of OPLS-DA of **(A)** P1, **(B)** P2, **(C)** P3 and **(D)** P4 compared with control group, respectively.

**Figure S13.** Volcano plots were used to select the candidate biomarkers. Acylcarnitines exhibited statistically significant changes after the administration of **(A)** P1, **(B)** P2, **(C)** P3, and **(D)** P4, respectively. The x-axis represents the multiplication relationship after logarithmic transformation, and the y-axis represents the *P* value after logarithmic transformation. The dots marked with blue indicate that the acylcarnitines significantly downregulated, the dots marked with red indicate that the acylcarnitines significantly upregulated and the green dots represent no significant acylcarnitines.

**Figure S14.** ROC analysis was used to show the sensitivity and specificity of the acylcarnitine data. Biomarkers reconfirm by ROC analysis of the plasma and brain acylcarnitine data. The reconfirmation of the potential biomarkers exhibited statistically significant changes after the administration of **(A)** P1, **(B)** P2, **(C)** P3, and **(D)** P4, respectively. The smaller the x-axis is, the higher the accuracy will be. The larger the y-axis is, the better the accuracy is. The AUC represents the accuracy of prediction. AUC > 0.8 indicated good predictive ability.

**Figure S15.** Result of hot plate test. **(A)** Analysis of differences between the P1-P4 groups and control group in hot plate reaction times before gavage. “ns” represents that the difference between the P1-P4 groups and the control group is not statistically significant. **(B)** Analysis of differences in hot plate reaction times before and after gavage in P1-P4 groups. **p* < 0.05, compared with before gavage. *****p* < 0.0001, compared with before gavage. **(C)** Analysis of differences between the P2-P4 groups and P1 group in hot plate reaction times after gavage. *****p* < 0.0001, compared with P1 group.

**Figure S16. (A)** Result of the OPLS-DA score scatter plots that show plasma and brain metabolic differences between the Pcom and the control group. **(B)** Result of the permutation test, the modeling effect of OPLS-DA of Pcom compared with the control group. Y-axis intercepts: R2 = (0.0, 0.823) and Q2 = (0.0, -1.05). **(C)** Volcano plot analysis of the plasma and brain acylcarnitine data. The x-axis represents the multiplication relationship after logarithmic transformation, and the y-axis represents the *P* value after logarithmic transformation. The green dots represent no significant metabolites. **(D)** ROC analysis was used to show the sensitivity and specificity of the acylcarnitine data.

**Table S1.** The gradient of mobile phase for the UHPLC-MS/MS analysis of acylcarnitines.

**Table S2.** MRM transition parameters for the acylcarnitines and their standards.

**Table S3.** Sensitivity and linear range of acylcarnitines.

**Table S4.** Data for accuracy, precision and ME of the method.

**Table S5.** Concentration of PHB in plasma (μg mL^-1^).

**Table S6.** Concentration of acylcarnitines (Mean ± SD) in different samples (ng mL^-1^).

**Table S7.** Associations between the plasma PHB and acylcarnitines in different samples (reported as *P*_trend_-value).

**Table S8.** The main parameters of the OPLS-DA model.

**Table S9.** VIP, -Log_10_ *P*-value and Log_2_ Fold Change in OPLS-DA model of first strategy.

**Table S10.** The data of the hot plate test.

**Table S11.** VIP, -Log_10_ *P*-value and Log_2_ Fold Change in OPLS-DA model of second strategy.

**Table S12.** The AUC values of potential biomarkers.

1. **Flat membrane liquid-phase micro-extraction for PHB extraction in plasma**

The device of flat membrane liquid-phase micro-extraction (FM-LPME) is shown in Fig. S1. Due to the high concentration of PHB in the plasma of poisoned rats, the sample solution was prepared by diluting the plasma with 12.5% NaCl in 10 mM HCl (DIC concentration was 50 ng mL^-1^) before sample pretreatment. In FM-LPME, 4 μL of the 2-nonanone was immobilized in the micropores of the polypropylene fiber flat membrane. The LPME was conducted from 800 μL of sample phase into 100 μL of acceptor phase (20 mM Na_3_PO_4_). The whole device was agitated by a MIC-100 constant temperature mixer (Hangzhou MIULAB Instrument Co. Ltd.) with a speed of 1250 rpm for 60 min. Immediately, the acceptor phase solution was collected for UHPLC-MS/MS analysis.


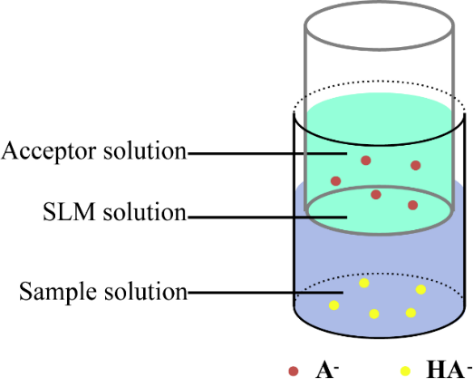


**Figure S1.** FM-LPME device.

1. **The optimization of the protein precipitation method for CAR extraction in plasma and brain**

For the organic solvent protein precipitation method, the protein precipitation effect of different organic solvents is different, and the extraction recovery rate of the target compound is also different, so we have optimized the organic solvent used for protein precipitation. Finally, ACN (containing 0.1% FA), which has a stronger ability to precipitate proteins, was selected as the solvent for protein precipitation.

We mainly compared the extraction effects of 8 different solvent systems on plasma and brain tissues. 8 different organic solvents mainly include ACN, MeOH, ACN/MeOH (50/50), ACN/ISO (50/50), ACN (with 0.1% FA), MeOH (with 0.1% FA), ACN/MeOH (50/50, with 0.1% FA), ACN/ISO (50/50, with 0.1% FA).

For plasma samples, take 100 μL of spiked artificial plasma (500 ng mL^-1^ for each analyte) into a 2 mL centrifuge tube, and then add 500 μL of the above solvent (4 parallel samples for each solvent) for protein precipitation. vortex for 30 s, the supernatant was collected by centrifugation, dried with nitrogen, reconstituted with 100 μL of ACN/H_2_O (90/10), and then analyzed by LC-MS/MS. The extraction recoveries of different solvents for each target were compared. The results are shown in Fig. S2.

**
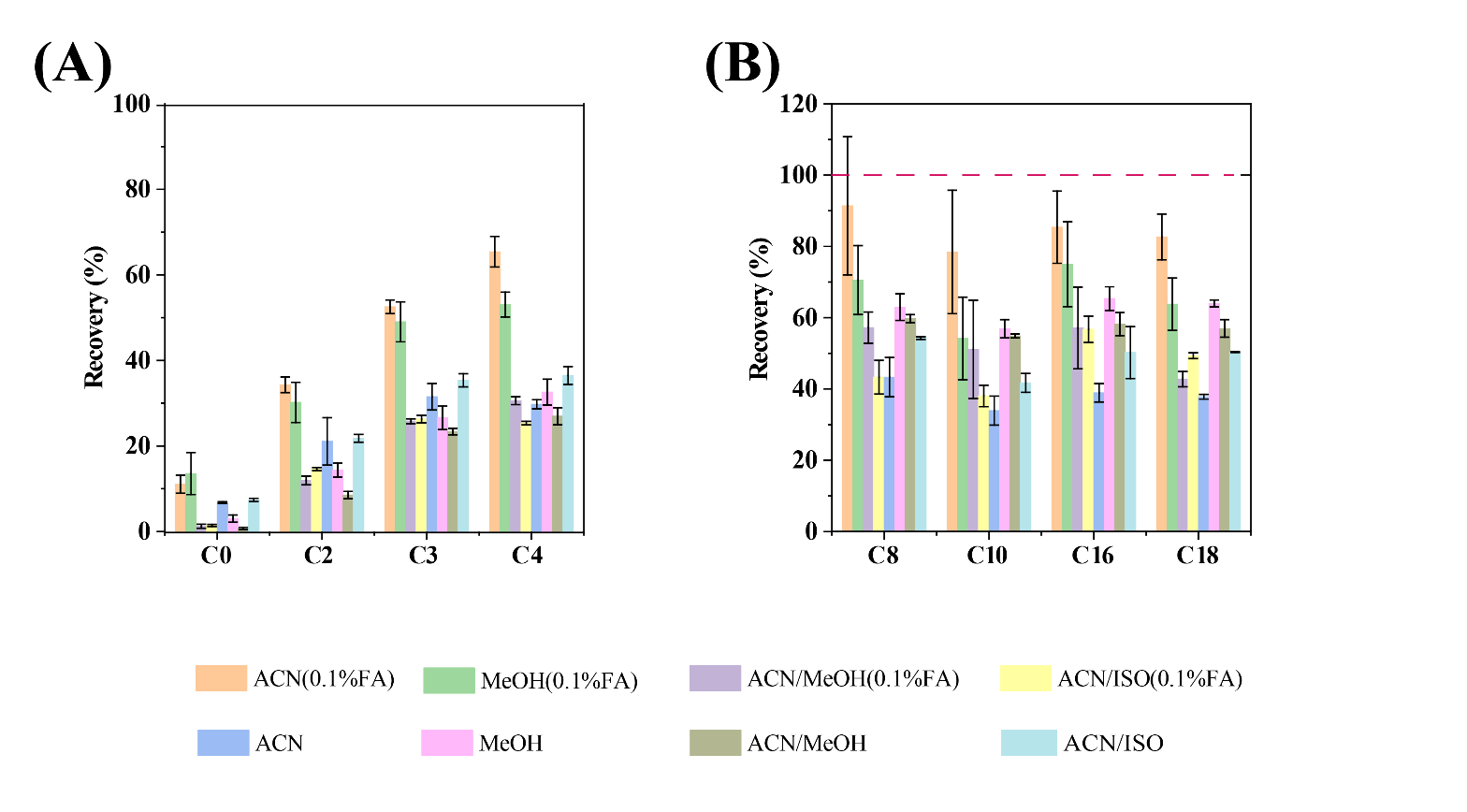
**

**Figure S2.** Recovery of protein precipitation extraction of **(A)** C0, C2, C3 and C4, **(B)** C8, C10, C16 and C18 from artificial plasma with different organic solvents.

For the protein precipitation of brain tissue, the concentration of each analyte is 500 ng mL^-1^ in the spiked brain tissue homogenate, and the extraction procedure is the same as that of plasma. The best organic solvent is selected by comparing the peak areas of the target compounds after extraction. The extraction results are shown in Fig. S3. The ordinate is the ratio of the peak area of the target extracted by each solvent to the peak area of the target extracted by ACN (0.1% FA).


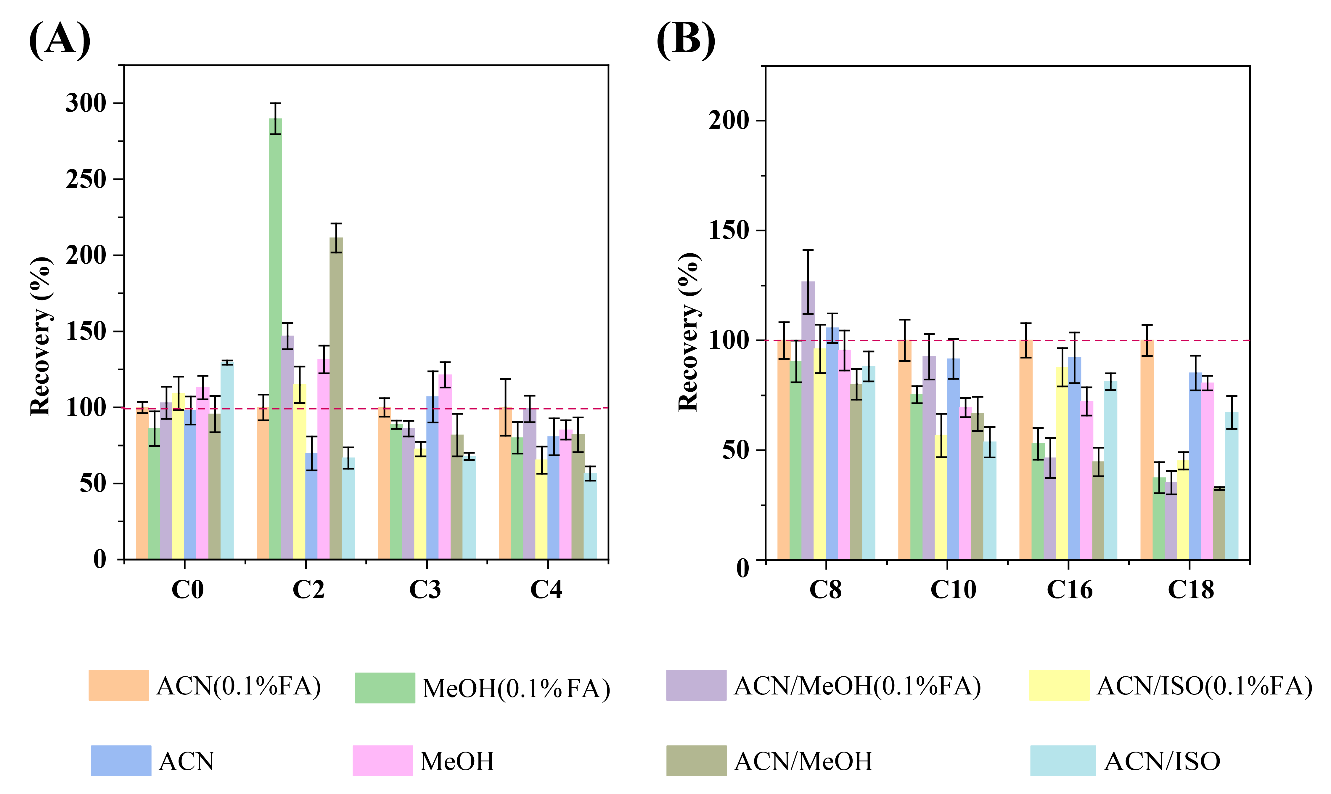


**Figure S3.** Recovery of protein precipitation extraction of **(A)** C0, C2, C3 and C4, **(B)** C8, C10, C16 and C18 from brain tissue with different organic solvents.

1. **The optimization of the condition of UHPLC-MS/MS for CAR analysis**

CARs are a family of compounds with simple aliphatic structures, chiral centers, and permanent positive charges, making them difficult to determine. A set of strategies were applied and the chromatographic and mass conditions were optimized to address these challenges in this study. UHPLC-MS analysis was conducted by using an Ultimate3000 UHPLC system, which includes a pump (LPG-3400RS), an autosampler (WPS-3000RS), a column oven (TCC-3000RC), and a TSQ Quantum Access MAX triple quadrupole Mass Spectrometry (Thermo Scientific, Waltham, MA, USA). HPLC system and the MS were controlled by Chromeleon client and Xcalibur software, respectively.

Acylcarnitines are mostly non-polar compounds, and we selected three different columns for comparison, including Hypersil GOLD C18 (2.1×100 mm, 3 µm, Thermo Fisher Scientific, USA), Acquity UPLC HSS T 3 (2.1×100 mm, 1.8 µm), Waters) and Acquity UPLC BEH Amide (2.1 × 100 mm, 1.7 µm, Waters) columns. Finally, the selected acylcarnitines were separated by Acquity UPLC BEH Amide column with satisfactory results. Therefore, chromatographic separations of acylcarnitines were performed on an ACQUITI UPLC BEH Amide column (1.7 μm, 2.1×150 mm, Waters Corporation, USA) combined with a ACQUITI UPLC BEH Amide pre-column (Waters Corporation, USA) with a column temperature of 45°C.

In the study of the retention time of the target compounds, it was found that the longer the carbon chain of acylcarnitines, the shorter the retention time on the chromatographic column, because the longer the carbon chain, the weaker the polarity and the lower the solubility in water. Compounds are less likely to enter the polar water layer and thus result in weaker retention times. To further improve the sensitivity and chromatographic performances of target analytes, we optimized the compositions of the mobile phase (Table S1). The mobile phase consisted of solvent A (1 mM NH_4_Ac and 0.1% FA in H_2_O/ACN (v/v = 95/5)) and solvent B (1 mM NH_4_Ac and 0.1% FA in H_2_O/ACN (v/v = 5/95)).

**Table S1** The gradient of mobile phase for the UHPLC-MS/MS analysis of CARs.

| Time/min | Flow rate (mL min^-1^) | A (%) | B (%) |
| --- | --- | --- | --- |
| 0 | 0.4 | 5 | 95 |
| 1.0 | 0.4 | 5 | 95 |
| 4.0 | 0.4 | 50 | 50 |
| 7.0 | 0.4 | 50 | 50 |
| 7.0 | 0.4 | 5 | 95 |
| 9.5 | 0.4 | 5 | 95 |

The mass spectrometer was operating at the following parameters: i) High-purity nitrogen (collision gas) served as sheath gas with a flow rate of 30 Arb and 10 Arb; ii) the spray voltage was 3.5 kV; iii) capillary temperature was set at 320 ℃; iv) evaporation temperature was set at 350°C; v) the electrospray ionization was under positive (ESI+). MRM monitoring conditions for carnitines and their standards were summarized in Table S2.

**Table S2** MRM transition parameters for the CARs and their standards.

| Analytes | Parent  (m/z) | Product  (m/z) | Collision energy (V) | Tube lens  (V) | IS |
| --- | --- | --- | --- | --- | --- |
| C0 | 162.1 | 60.4 | 16 | 71 | C2-*d*_3_ |
|  |  | 103.2* | 15 |  |  |
| C2 | 204.1 | 85.2* | 20 | 73 | C2-*d*_3_ |
|  |  | 99.1 | 18 |  |  |
| C3 | 218.1 | 85.2* | 17 | 66 | C2-*d*_3_ |
|  |  | 159.0 | 12 |  |  |
| C4 | 232.1 | 85.2* | 22 | 90 | C8-*d*_3_ |
|  |  | 173.1 | 13 |  |  |
| C8 | 288.2 | 85.2* | 21 | 89 | C8-*d*_3_ |
|  |  | 228.9 | 12 |  |  |
| C10 | 316.2 | 85.2* | 26 | 87 | C16-*d*_3_ |
|  |  | 257.0 | 16 |  |  |
| C16 | 400.2 | 85.2* | 28 | 120 | C16-*d*_3_ |
|  |  | 341.3 | 17 |  |  |
| C18 | 428.2 | 85.2* | 29 | 104 | C16-*d*_3_ |
|  |  | 369.3 | 17 |  |  |
| C2-d_3_ | 207.0 | 85.1* | 19 | 74 | / |
|  |  | 147.1 | 17 |  |  |
| C8-d_3_ | 291.7 | 85.1* | 22 | 77 | / |
|  |  | 228.9 | 13 |  |  |
| C16-d_3_ | 403.1 | 85.1* | 29 | 100 | / |
|  |  | 340.8 | 16 |  |  |

1. **Preparation of calibration standards and quality control solutions**

For calibration standard of PHB in plasma, PHB in sample solution was at final concentrations of 7.5, 15, 30, 75, 150, 300, and 750 ng mL^-1^ containing 75 ng mL^-1^ DIC. The QC level was 75 ng mL^-1^.

Since the concentration range of each acylcarnitines is different in different matrices, it is necessary to adjust the quantitative range of the calibration curve and the concentration of the quality control sample according to the specific matrix. To prepare the standard curves, 20 μL of specific working solutions and 4 μL (for plasma) or 8 μL (for brain) internal standard working solution were spiked into 100 μL artificial plasma or brain homogenates to generate calibration levels covering the specific ranges of analytes for different matrix (Table S3). The concentrations of the QC samples also need to be regulated according to different analytes in different matrices and be prepared in a real biological matrix. The corresponding QC levels added to real matrices for every analyte can be listed in Table S4.

1. **The method validation for CAR analysis**

Method validation of plasma used a surrogate matrix method, and brain tissue was validated by background subtraction.

**5.1. Limit of detection and quantification**

LOD is defined as the concentration level with a signal-to-noise ratio of 3, and the LOQ is defined as the concentration level with a signal-to-noise ratio of 10. For plasma samples, LOD and LOQ were obtained by using the corresponding analyte concentrations for S/N ratios of 3 and 10. While for brain tissue, since there is no real blank brain tissue and no surrogate matrix, the calibration standards (LOD and LOQ of the instrument) of the standard solution were used to evaluate its detection limit in brain tissue.

**5.2. Linearity range**

The validation of method linearity in plasma samples was conducted using the alternative matrix method. To determine the dynamic range of the method, four batches of calibration brain samples along with four blank samples were prepared and analyzed following the method mentioned above. The linearity range was investigated both in brain and plasma matrices. As the inherent existence of the endogenous substances in the matrix, the contribution of an endogenous level of each analyte as determined from the blank tissue must be subtracted. The peak-area ratio between the analyte and internal standard of the blank sample was subtracted from that of the corresponding spiked sample to give the increased peak-area ratio. The calibration curves were then constructed by plotting the increased peak-area ratio with the spiked concentration using linear regression for each compound, respectively.

**Table S3** Sensitivity and linear range of CARs.

| Analytes | Coefficient of determination (R^2^) | | Linear range  (ng mL^-1^) | | LOD  (ng mL^-1^) | | LOQ  (ng mL^-1^) | |
| --- | --- | --- | --- | --- | --- | --- | --- | --- |
|  | plasma | brain | plasma | brain | plasma | brain | plasma | brain |
| C0 | 0.9911 | 0.9902 | 50-10000 | 250-2000 | 0.8 | 1.0 | 2.7 | 3.4 |
| C2 | 0.9946 | 0.9921 | 50-10000 | 10-2000 | 0.1 | 0.5 | 0.4 | 1.7 |
| C3 | 0.9932 | 0.9910 | 10-1000 | 50-2000 | 0.1 | 0.9 | 0.4 | 3.0 |
| C4 | 0.9916 | 0.9963 | 10-1000 | 25-2000 | 0.2 | 0.6 | 0.7 | 2.0 |
| C8 | 0.9975 | 0.9987 | 5-2000 | 50-2000 | 0.4 | 0.2 | 1.4 | 0.7 |
| C10 | 0.9956 | 0.9910 | 5-2000 | 50-2000 | 1.8 | 0.7 | 6.1 | 2.4 |
| C16 | 0.9918 | 0.9901 | 5-2000 | 100-2000 | 0.4 | 0.1 | 1.5 | 0.4 |
| C18 | 0.9972 | 0.9916 | 5-2000 | 50-2000 | 0.2 | 0.7 | 0.7 | 2.4 |

**5.3. Accuracy, precision, and matrix effect**

Accuracy and precision were tested by testing QC samples with low, medium, and high concentrations (n = 6 at each concentration level). The precision is expressed as relative standard deviation (SD). The precision is expressed as relative standard deviation (RSD). The matrix effects (ME) were assessed by comparing the peak area of analyte from the spiked biological samples after extraction with the peak area of analyte from and the one of solvent standard at the same concentration (n = 6). Accuracy, precision, and ME for each analyte were calculated according to the following equations:

$$\text{SD}\text{（}\text{\%}\text{）}\text{=}\sqrt{\frac{\sum{\text{（}\bar{\text{X}}\text{-}\text{X}_{\text{i}}\text{）}}^{\text{2}}}{\text{n-1}}}\text{×100\%}$$

$$\text{RSD}\text{（}\text{\%}\text{）}\text{=SD/}\bar{\text{X}}\text{×100\%}\text{=}\sqrt{\frac{\sum{\text{（}\bar{\text{X}}\text{-}\text{X}_{\text{i}}\text{）}}^{\text{2}}}{\text{n-1}}\text{/}}\bar{\text{X}}\text{×100\%}$$

$$\text{ME }\left( \text{\%} \right)\text{=}\frac{\text{B-C}}{\text{A}}\text{×100\%}$$

Where A is the peak area of each analyte from standard solutions; B is the peak area of each analyte from spiked biological samples with the standards and IS after extraction; C is the peak area of each analyte from spiked biological samples with the standards and IS before extraction.

**Table S4** Data for accuracy, precision and ME of the method.

| Analytes | Spiked concentration (ng mL^-1^) | | Precision (%) | | Accuracy (%) | | ME (%) |
| --- | --- | --- | --- | --- | --- | --- | --- |
|  | plasma | brain | plasma | brain | plasma | brain | brain |
| C0 | 250.0 | 250.0 | 15.7 | 6.6 | 87.5 | 106.6 | - |
|  | 1000.0 | 500.0 | 10.7 | 13.9 | 111.2 | 101.7 | 96.0 |
|  | 5000.0 | 2000.0 | 5.6 | 3.0 | 111.2 | 101.3 | - |
| C2 | 100.0 | 50.0 | 18.2 | 8.0 | 109.6 | 82.6 | - |
|  | 1000.0 | 500.0 | 7.0 | 2.6 | 95.0 | 104.7 | 97.6 |
|  | 5000.0 | 1000.0 | 4.7 | 2.3 | 107.1 | 97.7 | - |
| C3 | 50.0 | 50.0 | 12.7 | 9.8 | 82.4 | 115.0 | - |
|  | 500.0 | 500.0 | 4.0 | 5.9 | 108.8 | 115.2 | 99.5 |
|  | 1000.0 | 1000.0 | 2.5 | 5.6 | 97.1 | 94.7 | - |
| C4 | 50.0 | 50.0 | 14.9 | 13.9 | 111.7 | 118.3 | - |
|  | 500.0 | 500.0 | 2.5 | 7.1 | 107.5 | 84.3 | 118.3 |
|  | 1000.0 | 1000.0 | 3.8 | 3.2 | 98.5 | 101.0 | - |
| C8 | 50.0 | 50.0 | 5.1 | 12.7 | 109.6 | 100.2 | - |
|  | 500.0 | 100.0 | 4.3 | 3.4 | 96.1 | 100.6 | 104.1 |
|  | 1000.0 | 500.0 | 4.1 | 2.8 | 97.4 | 100.2 | - |
| C10 | 50.0 | 50.0 | 10.1 | 12.3 | 112.5 | 87.7 | - |
|  | 500.0 | 100.0 | 5.9 | 10.5 | 102.7 | 118.9 | 87.1 |
|  | 1000.0 | 500.0 | 4.1 | 10.6 | 98.9 | 109.2 | - |
| C16 | 50.0 | 200.0 | 5.8 | 0.2 | 97.0 | 88.0 | - |
|  | 500.0 | 500.0 | 8.0 | 7.8 | 100.8 | 103.7 | 84.6 |
|  | 1000.0 | 2000.0 | 4.1 | 11.0 | 107.2 | 98.8 | - |
| C18 | 50.0 | 50.0 | 5.1 | 17.2 | 114.9 | 80.2 | - |
|  | 500.0 | 500.0 | 5.5 | 14.5 | 95.5 | 105.8 | 105.1 |
|  | 1000.0 | 1000.0 | 4.1 | 18.5 | 101.0 | 105.2 | - |

1. **The concentration of plasma PHB and CARs in the plasma and specific brain regions**

**Table S5** Concentration of PHB in plasma (μg mL^-1^).

| Gavage dose | 1 | 2 | 3 | 4 | 5 | 6 | 7 | Mean (SD) |
| --- | --- | --- | --- | --- | --- | --- | --- | --- |
| 1/8 LD_50_ (P1) | 478 | 525 | 553 | 492 | 392 | 606 | / | 507 (73) |
| 1/4 LD_50_ (P2) | 1030 | 930 | 772 | 1120 | 897 | 905 | / | 942 (120) |
| 1/2 LD_50_ (P3) | 1534 | 1348 | 673 | 675 | 1313 | 631 | 3330 | 1358 (947) |
| LD_50_ (P4) | 3153 | 4308 | 4434 | 3318 | 1710 | 1784 | 1745 | 2922 (1195) |

**Table S6** Concentration of acylcarnitines (Mean ± SD) in different samples (ng mL^-1^).

| Samples | Analytes | Control | 1/8 LD_50_ | 1/4 LD_50_ | 1/2 LD_50_ | LD_50_ |
| --- | --- | --- | --- | --- | --- | --- |
| Plasma | C0 | 3874.10±2666.70 | 6919.30±3616.80 | 1529.30±487.60 | 4830.30±2610.50 | 3550.30±3223.00 |
|  | C2 | 5523.70±1943.50 | 4113.60±1616.30 | 2682.60±704.30 | 2798.30±1110.90 | 2490.80±1507.60 |
|  | C3 | 283.70±166.30 | 280.50±107.90 | 56.30±11.40 | 172.30±110.50 | 93.80±77.90 |
|  | C4 | - | - | - | - | - |
|  | C8 | 100.70±113.00 | 12.70±9.40 | 23.70±16.50 | 32.90±52.10 | 13.00±14.10 |
|  | C10 | 28.40±19.00 | 7.60±5.20 | 2.80±2.00 | 6.60±4.30 | 1.60±0.80 |
|  | C16 | 165.30±23.00 | 73.10±16.20 | 157.40±82.20 | 120.00±56.50 | 126.70±31.40 |
|  | C18 | 36.70±6.20 | 18.30±5.90 | 41.40±14.80 | 29.90±6.20 | 33.20±6.00 |
| Hippocampus | C0 | 3643.80±564.20 | 5034.80±2330.60 | 3311.00±1003.60 | 8137.20±3800.60 | 5418.80±1790.60 |
|  | C2 | 1148.40±566.80 | 1196.80±376.00 | 1696.40±606.60 | 2147.20±616.00 | 1353.80±406.20 |
|  | C3 | 166.40±180.80 | 0.00±0.00 | 63.80±77.40 | 502.60±312.00 | 194.00±96.20 |
|  | C4 | 128.20±20.40 | 116.60±2.20 | 125.20±6.40 | 117.40±3.40 | 117.20±1.80 |
|  | C8 | 80.00±28.60 | 59.20±0.80 | 59.80±1.40 | 59.20±0.40 | 59.60±2.20 |
|  | C10 | 75.80±21.80 | 60.60±1.40 | 60.20±0.20 | 60.00±0.20 | 60.40±1.00 |
|  | C16 | 543.80±279.40 | 772.40±113.40 | 742.80±352.20 | 768.40±146.20 | 758.00±292.40 |
|  | C18 | 75.40±18.40 | 77.00±29.40 | 79.00±32.40 | 105.80±28.20 | 90.40±50.40 |
| Frontal lobe | C0 | 3430.80±974.00 | 4847.00±2923.80 | 4026.20±2904.20 | 6137.40±3588.00 | 3937.20±601.20 |
|  | C2 | 1294.20±512.00 | 1138.40±538.20 | 1203.20±469.60 | 1434.60±555.60 | 779.20±188.60 |
|  | C3 | 86.80±24.20 | - | 57.60±16.00 | 249.80±263.60 | 225.80±88.60 |
|  | C4 | 118.40±4.00 | 116.00±2.40 | 119.80±5.40 | 116.80±2.40 | 117.80±3.80 |
|  | C8 | 61.80±4.40 | 59.20±0.60 | 59.20±0.60 | 58.80±0.20 | 59.80±1.00 |
|  | C10 | 61.20±2.80 | 60.20±0.20 | 60.00±0.20 | 60.00±0.20 | 60.20±0.40 |
|  | C16 | 443.80±242.60 | 472.20±108.80 | 652.40±536.60 | 465.80±171.00 | 306.80±89.40 |
|  | C18 | 39.20±16.40 | 49.40±29.20 | 63.60±58.20 | 59.80±30.60 | 23.40±25.60 |
| Striatum | C0 | 3778.40±830.20 | 4363.60±762.00 | 3515.00±664.80 | 20106.40±30538.40 | 5051.60±1589.60 |
|  | C2 | 1106.80±427.00 | 1438.40±326.80 | 1581.60±414.80 | 2440.20±1170.20 | 1320.20±403.80 |
|  | C3 | 65.80±62.80 | - | 47.00±65.60 | 1663.40±2329.80 | 315.60±246.40 |
|  | C4 | 116.20±3.20 | 118.00±2.60 | 126.20±4.40 | 119.60±4.40 | 119.20±4.20 |
|  | C8 | 47.40±26.40 | 60.00±0.80 | 60.00±1.60 | 59.20±0.60 | 59.00±0.40 |
|  | C10 | 60.80±1.40 | 61.20±1.60 | 59.80±0.20 | 60.40±0.40 | 60.00±0.20 |
|  | C16 | 1096.40±726.40 | 878.80±273.60 | 773.80±283.40 | 1295.60±723.20 | 873.40±317.40 |
|  | C18 | 98.40±128.00 | 76.40±34.80 | 79.40±46.00 | 149.60±103.80 | 111.20±58.60 |
| Brainstem | C0 | 4601.00±335.60 | 5823.80±1810.40 | 3909.60±490.80 | 7212.20±2319.60 | 6717.40±1812.40 |
|  | C2 | 923.80±281.40 | 1076.40±357.60 | 1451.60±391.00 | 1415.40±327.60 | 909.80±182.40 |
|  | C3 | 78.40±105.60 | 0.00±0.00 | 78.40±59.80 | 449.40±241.00 | 426.20±551.20 |
|  | C4 | 120.80±7.80 | 117.80±3.00 | 128.40±12.80 | 119.40±3.60 | 115.40±3.40 |
|  | C8 | 68.80±17.00 | 59.20±0.40 | 59.00±0.60 | 59.80±1.80 | 58.80±0.20 |
|  | C10 | 72.20±20.40 | 61.20±1.20 | 60.00±0.20 | 60.40±0.60 | 60.20±0.20 |
|  | C16 | 1433.20±731.80 | 1594.00±416.60 | 1412.20±612.40 | 1071.80±478.60 | 1344.80±312.80 |
|  | C18 | 145.60±69.80 | 156.60±46.20 | 144.60±67.40 | 113.20±77.60 | 164.00±32.60 |

-note：not detected

1. **The cluster analysis of frontal lobe, striatum and brainstem**


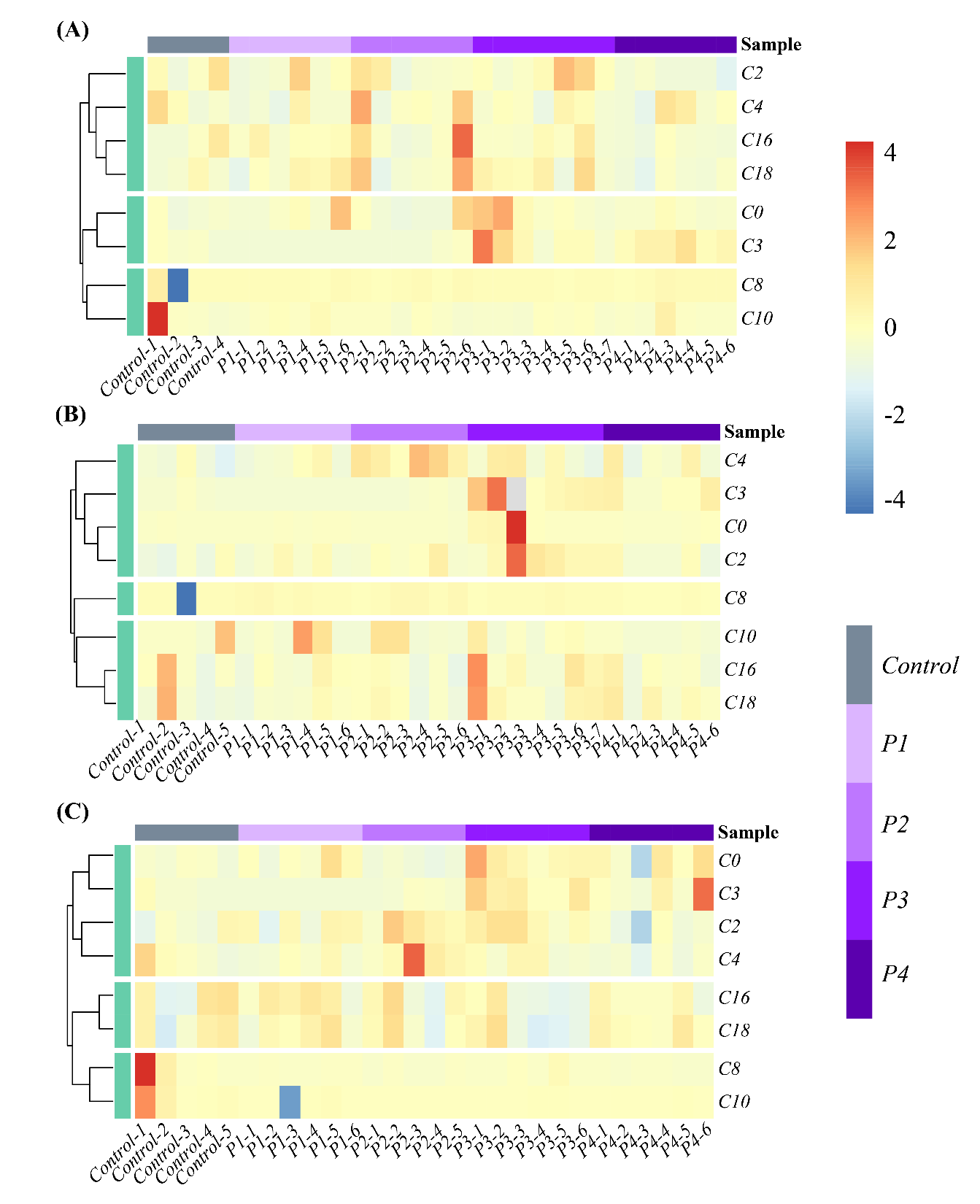


**Figure S4.** Heatmap and hierarchical clustering of **(A)** frontal lobe **(B)** striatum and **(C)** brainstem in the profile of CARs in acute PHB poisoning model for P1, P2, P3 and P4, compared with controls. Control-1 to 5 and P1-1 to P4-6 are the sample numbers of the control group and experimental groups, respectively.

1. **Correlation between PHB and CARs in plasma and brain**

Many researches have demonstrated that barbiturates exposure could affect the level of serum CARs, but CAR levels were primarily measured in serum instead of specific brain regions. The relationship between plasma PHB and broad-spectrum CAR changes in specific brain regions remained unclear. In order to clarify this, the effects of PHB poisoning on CAR metabolism in brain tissues were explored. For the brainstem, none of the tested CARs was significantly associated with PHB exposure (Table S7 and Fig. S9). However, for the other three brain regions, some CARs showed significantly associated with PHB exposure. The detailed information was given in the following text.

It is seen in Table S7, the *P*_trend_-values for C0 and C3 were all less than 0.05 (C0-*P*_trend_ = 0.03 and C3-*P*_trend_ = 0.01), indicating the plasma PHB in the 2nd, 3rd, and 4th quartiles had higher C0 and C3 concentrations in hippocampus than in the 1st quartile. This result was confirmed in Fig. S6, where C2 (*P*_trend_ = 0.003) and C3 (*P*_trend_ = 0.001) in hippocampus showed an increasing trend with increasing PHB concentrations. However, C2, C4, C8, C10, C16, and C18 in hippocampus were not significantly associated with PHB exposure (Table S7 and Fig. S6).

**Table S7** Associations between the concentration of PHB and acylcarnitines in different samples (reported as *P*_trend_-value).

| Sample | PHB quartiles | 1st quartile  (< 492.09) | 2nd quartile  (492.09-772.19) | 3rd quartile  (772.20-1534.09) | 4th quartile  (≥ 1534.10) | *P*_trend_ | |
| --- | --- | --- | --- | --- | --- | --- | --- |
| Plasma | C0 | 4566.15(3248.80,5833.16) | 6906.89(3652.93,7579.50) | 1640.57(792.13,2837.57) | 2549.72(1940.19,4041.00) | 0.24 | |
|  | C2 | 4120.42(3566.17,6534.95) | 3681.12(2626.24,4185.42) | 1996.48(1413.77,2897.98) | 2086.27(1814.33,3770.34) | **0.003** | |
|  | C3 | 256.55(209.94,379.74) | 235.53(127.71,373.69) | 59.27(26.22,90.40) | 62.49(43.50,133.91) | **0.001** | |
|  | C8 | 45.70(10.84,79.68) | 8.46(0.28,18.68) | 12.06(1.08,25.36) | 7.59(1.43,16.07) | 0.11 | |
|  | C10 | 14.62(8.79,29.52) | 5.04(3.57,6.72) | 2.75(2.17,7.59) | 1.39(1.13,2.59) | **0.002** | |
|  | C16 | 151.44(109.15,165.85) | 90.68(77.06,108.05) | 113.18(88.70,237.36) | 116.27(110.60,122.97) | 0.74 | |
|  | C18 | 34.37(25.10,38.71) | 25.33(17.19,29.41) | 39.81(27.84,46.47) | 29.21(28.25,34.92) | 0.32 | |
| Hippocampus | C0 | 3863.59(3390.67,4334.57) | 4815.64(3874.37,5518.68) | 3795.75(3584.35,5192.14) | 5521.61(4800.47,8936.40) | **0.03** | |
|  | C2 | 1126.98(948.97,1372.32) | 1562.58(1357.96,1931.72) | 1878.22(1535.47,2388.62) | 1512.88(1256.18,1624.19) | 0.32 | |
|  | C3 | 1.27(1.27,99.58) | 1.27(1.27,131.12) | 134.38(37.09,381.82) | 193.23(137.91,469.42) | **0.01** | |
|  | C4 | 117.18(114.10,124.52) | 115.52(114.82,119.57) | 119.27(119.15,130.01) | 118.50(116.82,119.18) | 0.43 | |
|  | C8 | 59.40(44.09,65.42) | 58.91(58.66,59.23) | 58.83(58.60,59.36) | 58.81(58.67,59.22) | 0.72 | |
|  | C10 | 60.42(60.04,71.35) | 59.80(30.40,59.92) | 59.91(30.39,60.11) | 59.99(59.93,60.07) | 0.43 | |
|  | C16 | 701.81(428.75,819.57) | 714.06(673.68,796.91) | 808.55(727.97,943.94) | 638.81(598.61,801.42) | 0.35 | |
|  | C18 | 74.75(62.29,98.56) | 57.54(48.18,86.16) | 90.76(67.10,112.28) | 110.48(76.60,123.75) | 0.14 | |
| Frontal  lobe | C0 | 3260.72(3124.85,3912.79) | 3931.93(2979.09,5861.86) | 3943.18(3213.42,10816.76) | 3818.63(3746.27,4207.15) | 0.51 | |
|  | C2 | 1016.47(817.00,1486.13) | 1130.17(980.61,1232.29) | 1259.05(912.75,1707.33) | 913.45(809.86,1133.31) | 0.47 | |
|  | C3 | 1.27(1.27,70.36) | 35.42(1.27,71.88) | 143.90(1.27,241.70) | 197.15(142.59,231.82) | **0.009** | |
|  | C4 | 116.21(115.62,118.76) | 116.37(115.24,117.77) | 117.07(115.58,118.06) | 117.21(115.08,118.46) | 0.99 | |
|  | C8 | 59.24(58.70,59.57) | 58.88(58.67,59.55) | 59.03(58.80,59.81) | 59.04(58.94,60.05) | 0.23 | |
|  | C10 | 60.06(59.91,60.20) | 60.01(59.84,60.14) | 59.98(59.90,60.07) | 59.97(59.94,60.00) | 0.22 | |
|  | C16 | 432.37(361.67,534.37) | 433.96(331.18,520.46) | 390.64(329.98,511.48) | 355.53(288.57,465.26) | 0.54 | |
|  | C18 | 40.01(30.00,61.67) | 37.27(31.10,78.26) | 46.29(25.36,82.29) | 36.09(28.19,47.23) | 0.74 | |
| Striatum | C0 | 3879.39(3416.71,4459.97) | 4855.14(4074.46,5270.54) | 4927.01(3363.82,7777.53) | 4679.68(3993.53,7611.02) | 0.06 | |
|  | C2 | 1209.95(885.17,1467.17) | 1919.66(1376.03,2331.14) | 1606.48(1315.97,1777.31) | 1891.09(1169.55,2117.71) | 0.07 | |
|  | C3 | 22.25(1.27,56.61) | 81.25(1.27,274.80) | 164.21(25.16,685.53) | 253.13(118.44,659.20) | 0.06 | |
|  | C4 | 116.52(115.72,120.35) | 117.30(116.47,120.63) | 124.07(121.84,125.35) | 117.83(117.44,120.75) | 0.22 | |
|  | C8 | 59.18(59.00,59.78) | 59.50(59.03,60.53) | 58.97(58.79,60.13) | 58.70(58.65,59.01) | 0.23 | |
|  | C10 | 60.15(60.09,61.89) | 60.08(59.93,60.22) | 59.99(59.82,60.71) | 59.93(59.91,60.17) | **0.045** | |
|  | C16 | 884.27(660.62,1060.49) | 886.04(736.73,1001.02) | 931.24(766.60,1205.94) | 862.11(811.16,1179.09) | 0.95 | |
|  | C18 | 57.98(33.55,85.98) | 87.16(72.79,96.69) | 127.28(85.17,147.06) | 73.47(71.12,93.84) | 0.68 | |
| Brainstem | C0 | 4878.16(4624.68,5328.93) | 5454.45(4833.41,6215.52) | 4691.35(3942.62,6993.88) | 4593.36(4406.23,6719.65) | 0.88 |  |
|  | C2 | 979.61(750.40,1288.99) | 1262.50(1245.44,1534.37) | 1117.41(944.09,1394.63) | 1307.94(935.60,1612.70) | 0.59 |  |
|  | C3 | 1.27(1.27,65.51) | 42.77(1.27,347.80) | 136.95(81.58,330.51) | 131.92(83.10,231.96) | 0.25 |  |
|  | C4 | 117.85(116.35,121.40) | 120.30(116.52,122.50) | 119.82(117.18,124.53) | 115.65(114.24,117.59) | 0.53 |  |
|  | C8 | 59.52(59.19,61.99) | 59.45(58.93,59.75) | 58.61(58.54,58.85) | 58.74(58.67,58.82) | 0.09 |  |
|  | C10 | 61.81(60.47,64.26) | 60.00(59.97,60.09) | 60.08(59.99,60.20) | 60.11(60.01,60.23) | 0.47 |  |
|  | C16 | 1730.86(1107.81,2014.80) | 935.55(875.46,1795.12) | 1182.88(822.16,1571.63) | 1619.71(1319.88,1949.07) | 0.84 |  |
|  | C18 | 181.61(114.57,190.21) | 120.44(84.04,154.49) | 142.66(105.74,162.62) | 182.04(148.61,220.96) | 0.28 |  |

It is seen in Table S7 that the *P*_trend_-value for C3 was less than 0.05 (C3-*P*_trend_ = 0.009) indicating that plasma PHB in the 2nd, 3rd and 4th quartiles had higher C3 concentration in frontal lobe than in the 1st quartile. As can be seen that C3-*P*_trend_ = 0.009 (in Fig. S7B) in frontal lobe also show an increasing trend with increasing PHB concentrations. On the contrary, C0, C2, C4, C8, C10, C16, and C18 in frontal lobe were not significantly associated with PHB exposure (Table S7 and Fig. S7).

It is seen in Table S7 that the *P*_trend_-value for C10 was less than 0.05 (C10-*P*_trend_ = 0.045) indicating that plasma PHB in the 2nd, 3rd, and 4th quartiles had lower C10 concentrations in striatum than in the 1st quartile. In addition, C0, C2, C3, C4, C8, C16, and C18 in striatum were not significantly associated with PHB exposure (Table S7 and Fig. S8).

In short, it is generally seen that C0 and short-chain acylcarnitines increased in the brain, the medium-chain acylcarnitine levels were more constant and long-chain acylcarnitines changed randomly. Our results also showed that in most instances, the alterations of CARs in brain were not consistent with those observed in the plasma (see the CAR concentration changes in Table S7 and Fig. S5-S9). From the perspective of the results that reached statistical significance (*P*_trend_ < 0.05), in contrast to the decrease of plasma C2, C3, and C10 with the increase of PHB, an increase of C0 and C3 in hippocampus and an increase of C3 in frontal lobe were found. The above findings suggested that PHB poisoning affects plasma and brain CARs differently.

1. **Plasma CARs inadequately reflect brain CARs metabolism**

**9.1 Correlation between the levels of acylcarnitines in plasma & specific brain regions and quartiles of plasma PHB concentration**

**Plasma:**


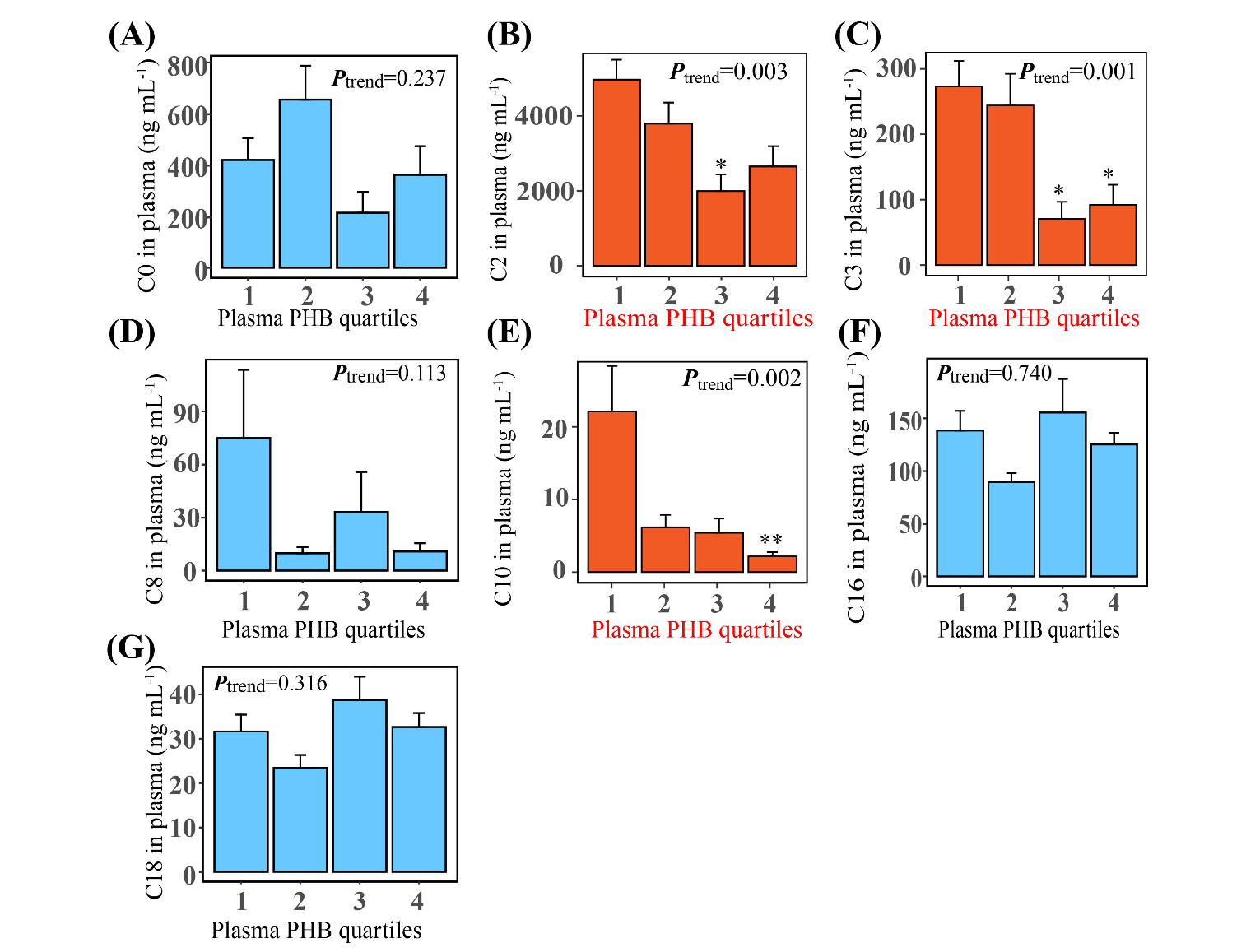


**Figure S5.** Levels of **(A)** C0, **(B)** C2, **(C)** C3, **(D)** C8, **(E)** C10, **(F)** C16 and **(G)** C18 (ng mL^-1^) in plasma by quartiles of plasma PHB concentration. Range of PHB quartiles [μg mL^-1^]: 1st, < 492.09; 2nd, 492.09-772.19; 3rd, 772.20-1534.09; 4th, ≥ 1534.10. Bars correspond to estimated mean values of acylcarnitines. Whiskers indicate standard error. *P*_trend_, *P*-value for linear trend across quartiles of PHB. The acylcarnitines that showed significantly decreasing trend with increasing PHB concentrations. were highlighted with red color.**Hippocampus:**


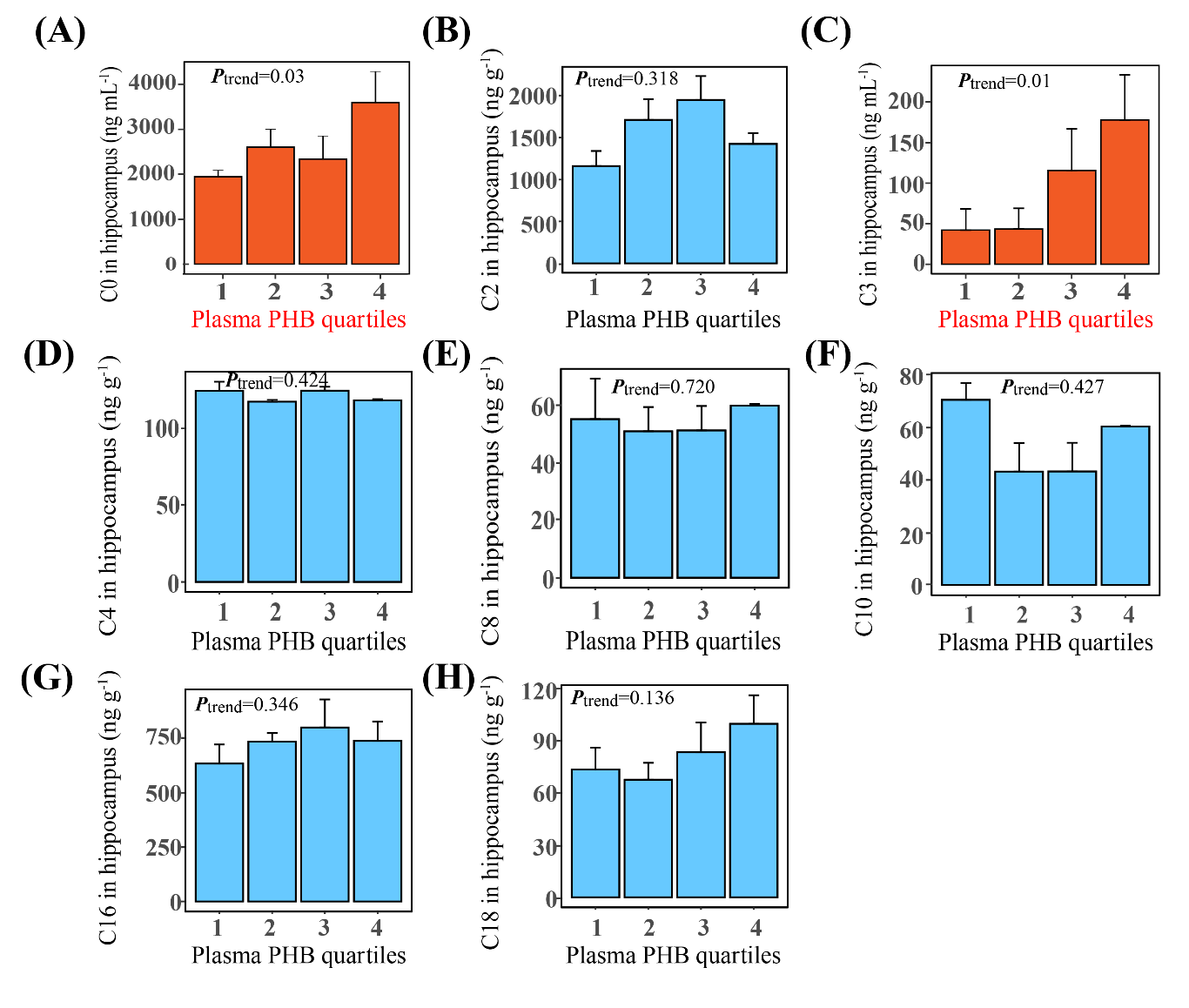


**Figure S6.** Levels of **(A)** C0, **(B)** C2, **(C)** C3, **(D)** C4, **(E)** C8, **(F)** C10, **(G)** C16 and **(H)** C18 (ng g^-1^) in hippocampus by quartiles of plasma PHB concentration. Range of PHB quartiles [μg mL^-1^]: 1st, < 492.09; 2nd, 492.09-772.19; 3rd, 772.20-1534.09; 4th, ≥ 1534.10. Bars correspond to estimated mean values of acylcarnitines. Whiskers indicate standard error. *P*_trend_, *P*-value for linear trend across quartiles of PHB. The acylcarnitines that showed significantly increasing trend with increasing PHB concentrations were highlighted with red color.

**Frontal lobe:**


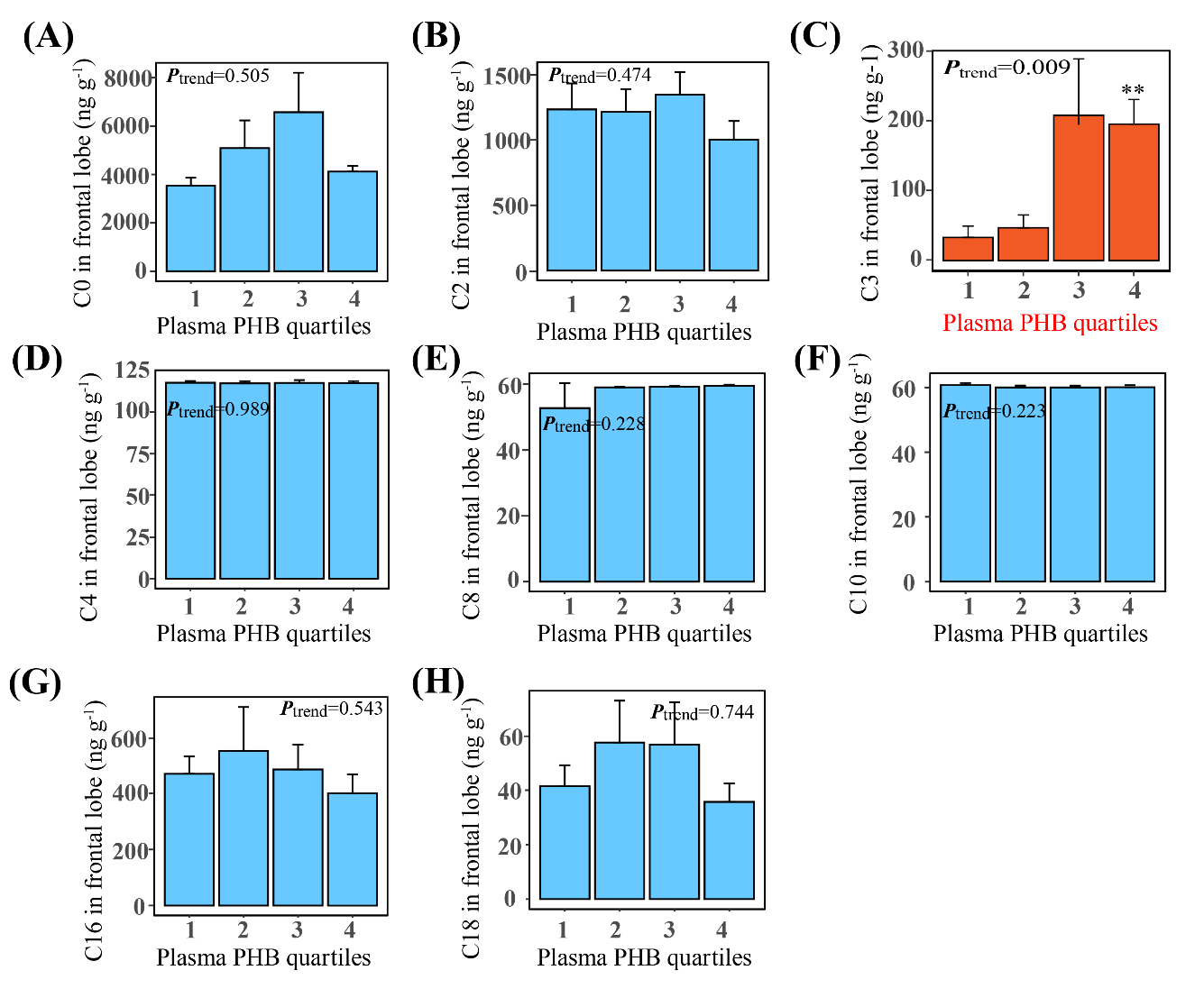


**Figure S7.** Levels of **(A)** C0, **(B)** C2, **(C)** C3, **(D)** C4, **(E)** C8, **(F)** C10, **(G)** C16 and **(H)** C18 (ng g^-1^) in frontal lobe by quartiles of plasma PHB concentration. Range of PHB quartiles [μg mL^-1^]: 1st, < 492.09; 2nd, 492.09-772.19; 3rd, 772.20-1534.09; 4th, ≥ 1534.10. Bars correspond to estimated mean values of acylcarnitines. Whiskers indicate standard error. *P*_trend_, *P*-value for linear trend across quartiles of PHB. The acylcarnitines that showed significantly increasing trend with increasing PHB concentrations were highlighted with red color.

**Striatum:**


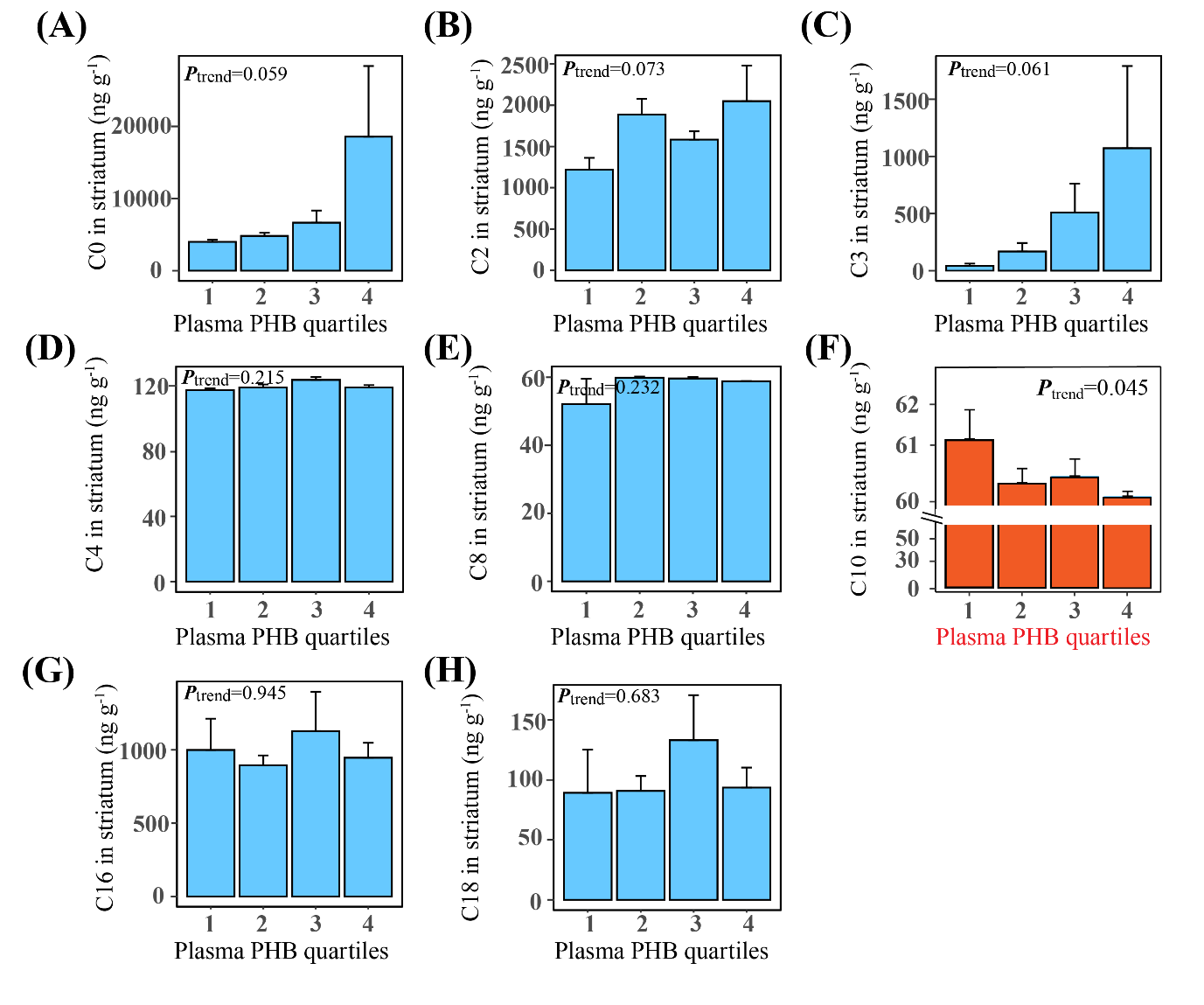


**Figure S8.** Levels of Levels of **(A)** C0, **(B)** C2, **(C)** C3, **(D)** C4, **(E)** C8, **(F)** C10, **(G)** C16 and **(H)** C18 (ng g^-1^) in striatum by quartiles of plasma PHB concentration. Range of PHB quartiles [μg mL^-1^]: 1st, < 492.09; 2nd, 492.09-772.19; 3rd, 772.20-1534.09; 4th, ≥ 1534.10. Bars correspond to estimated mean values of acylcarnitines. Whiskers indicate standard error. *P*_trend_, *P*-value for linear trend across quartiles of PHB. The acylcarnitines that showed significantly decreasing trend with increasing PHB concentrations were highlighted with red color.

**Brainstem:**


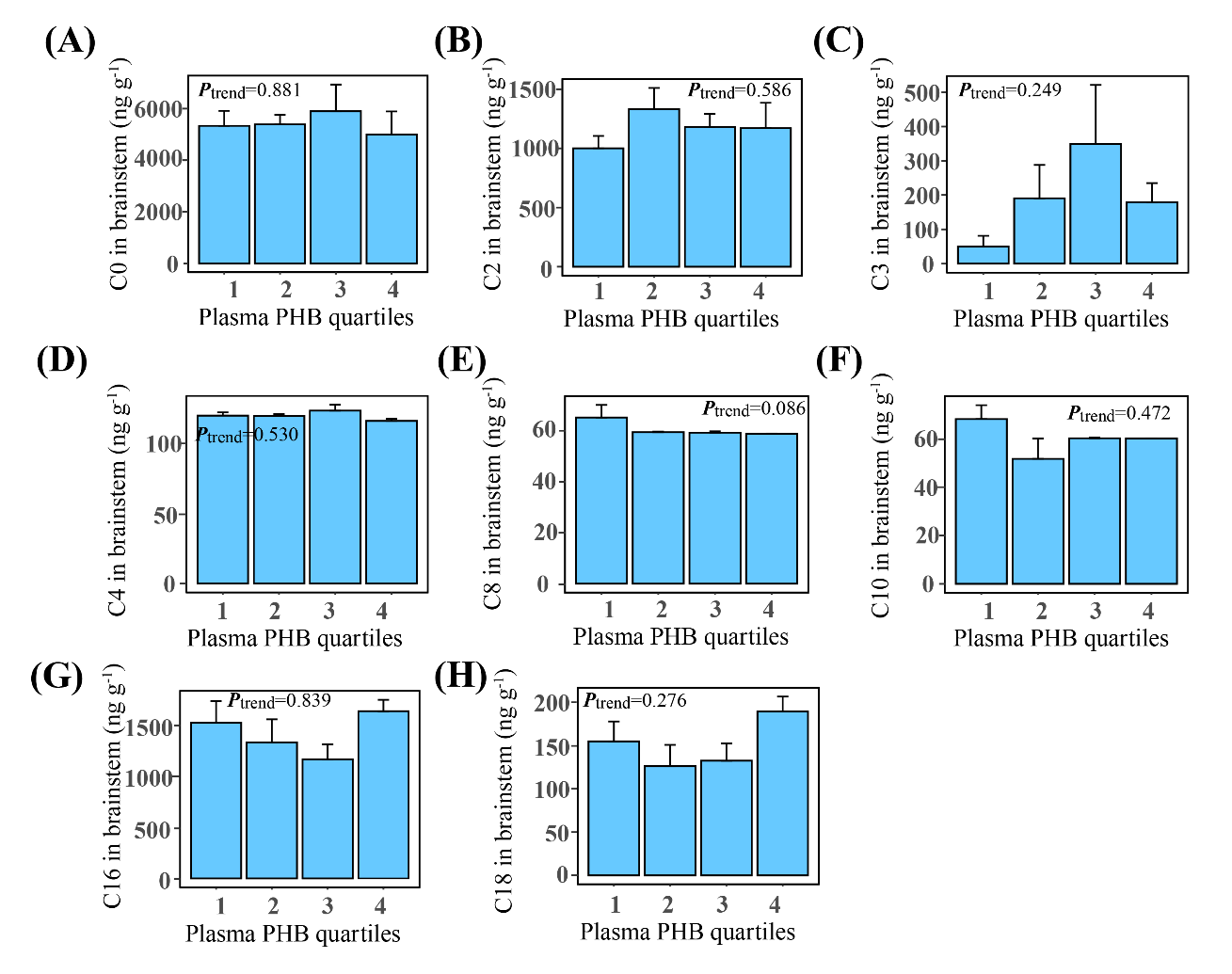


**Figure S9.** Levels of Levels of **(A)** C0, **(B)** C2, **(C)** C3, **(D)** C4, **(E)** C8, **(F)** C10, **(G)** C16 and **(H)** C18 (ng g^-1^) in brainstem by quartiles of plasma PHB concentration. Range of PHB quartiles [μg mL^-1^]: 1st, < 492.09; 2nd, 492.09-772.19; 3rd, 772.20-1534.09; 4th, ≥ 1534.10. Bars correspond to estimated mean values of acylcarnitines. Whiskers indicate standard error. *P*_trend_, *P*-value for linear trend across quartiles of PHB. No acylcarnitines showed significantly decreasing trend with increasing PHB concentrations in brainstem.

**9.2 Correlation between acylcarnitines in plasma and acylcarnitines in brain**

**
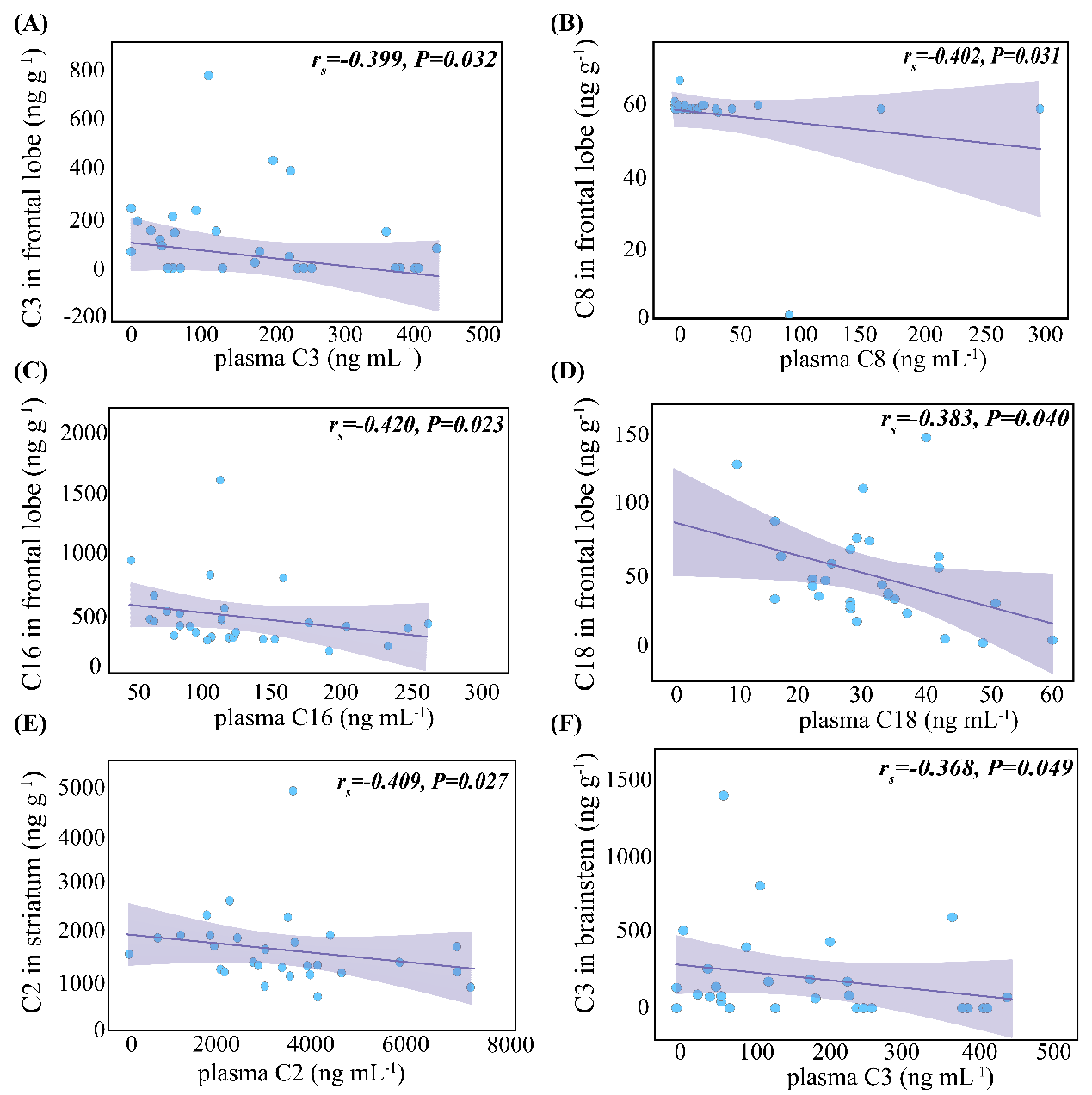
**

**Figure S10.** Scattering plot for the correlations of **(A)** C3, **(B)** C8, **(C)** C16, **(D)** C18, **(E)** C2 and **(F)** C3 between plasma and specific brain regions. “*r*_s_” indicates Spearman correlation coefficient. Correlation is significant at *p* < 0.05 (2-tailed). Note that the y-axis is the levels of acylcarnitines (ng g^-1^) in the specific brain region samples and the x-axis is the plasma acylcarnitine concentration (ng mL^-1^).

1. **The hot plate experiment data**

**Table S8** The data of the hot plate test.

| Sample | before gavage (s) | after gavage (s) |
| --- | --- | --- |
| Control-1 | 34 | - |
| Control-2 | 15 | - |
| Control-4 | 26 | - |
| Control-5 | 13 | - |
| Control-6 | 18 | - |
| P1-1 | 18 | 44 |
| P1-2 | 4 | 10 |
| P1-3 | 4 | 40 |
| P1-4 | 8 | 40 |
| P1-5 | 20 | 13 |
| P1-6 | 5 | 30 |
| P2-1 | 6 | 60 |
| P2-2 | 12 | 60 |
| P2-3 | 11 | 60 |
| P2-4 | 4 | 60 |
| P2-5 | 11 | 60 |
| P2-6 | 6 | 60 |
| P2-7 | 8 | 60 |
| P3-1 | 18 | 60 |
| P3-2 | 13 | 60 |
| P3-3 | 10 | 60 |
| P3-4 | 12 | 60 |
| P3-5 | 12 | 60 |
| P3-6 | 15 | 60 |
| P3-7 | 8 | 60 |
| P4-1 | 19 | 60 |
| P4-2 | 9 | 60 |
| P4-3 | 16 | 60 |
| P4-4 | 12 | 60 |
| P4-6 | 6 | 60 |
| P4-7 | 6 | 60 |

**
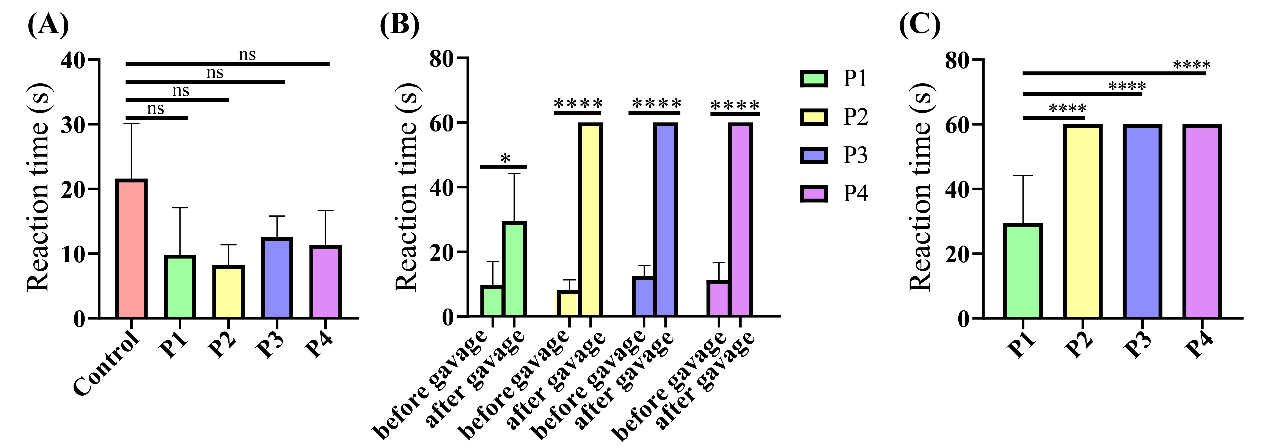
**

**Figure S11.** Result of hot plate test. **(A)** Analysis of differences between the P1-P4 groups and control group in hot plate reaction times before gavage. “ns” represents that the difference between the P1-P4 groups and the control group is not statistically significant. **(B)** Analysis of differences in hot plate reaction times before and after gavage in P1-P4 groups. **p* < 0.05, compared with before gavage. **p* < 0.0001, compared with before gavage. **(C)** Analysis of differences between the P2-P4 groups and P1 group in hot plate reaction times after gavage. **p* < 0.0001, compared with P1 group.

1. **The main parameters of the OPLS-DA model (three indicators of** **first strategy and second strategy)**

**Table S9** The main parameters of the OPLS-DA model.

| Strategy | Model | Group | R2X(cum) | R2Y(cum) | Q2Y(cum) |
| --- | --- | --- | --- | --- | --- |
| First strategy | OPLS-DA | P1 | 0.599 | 0.993 | 0.703 |
|  |  | P2 | 0.583 | 0.986 | 0.774 |
|  |  | P3 | 0.497 | 0.985 | 0.784 |
|  |  | P4 | 0.709 | 0.993 | 0.792 |
| Second strategy | OPLS-DA | Pcom | 0.467 | 0.975 | 0.643 |

1. **The permutation test of OPLS-DA model in first strategy**

It is easy to over-fit when using supervised learning methods for analysis. To this end, it is necessary to use the permutation test to examine the modeling effect of OPLS-DA. By examining the intercept of the fitting straight line composed of R2 and Q2 calculated values of all samples on the Y coordinate axis, it indicates the reliability and overfitting degree of the model. The results satisfy that the crossing point of the regression line of Q2 on the y-ordinate is less than 0 (Fig. S15a-d). Therefore, the modeling results of OPLS-DA are acceptable.


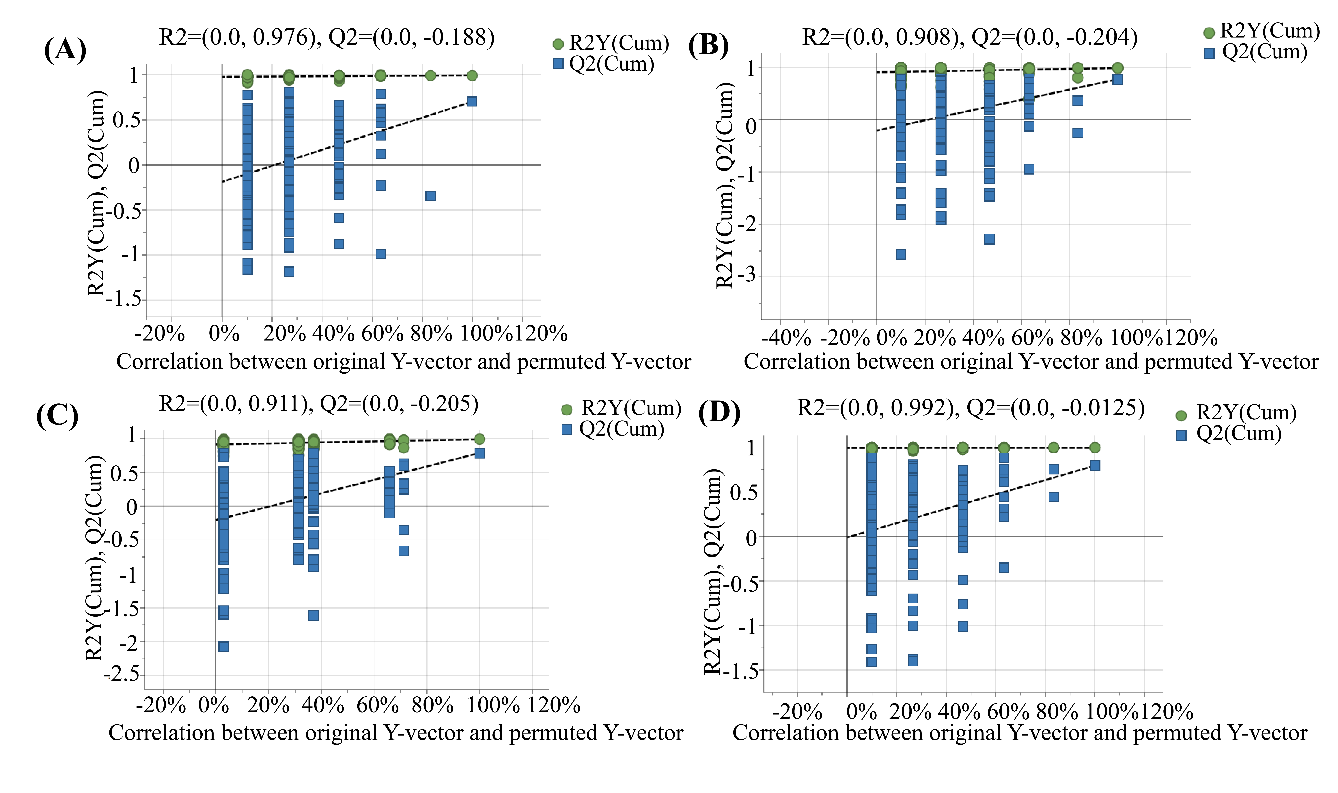


**Figure S12.** Result of the permutation test, the modeling effect of OPLS-DA of **(A)** P1, **(B)** P2, **(C)** P3 and **(D)** P4 compared with control group, respectively.

1. **The values of VIP, -Log_10_ *P*-value and Log_2_ Fold Change in the OPLS-DA model (first strategy)**

**Table S10** The values of VIP, -Log_10_ *P*-value and Log_2_ Fold Change in the OPLS-DA model of first strategy.

| First strategy | Sample ID | VIP | *P*-value | -Log_10_ *P*-value | Fold Change | Log_2_ Fold Change |
| --- | --- | --- | --- | --- | --- | --- |
| P1 | plasma-C0 | 1.01 | 0.15 | 0.81 | 1.79 | 0.84 |
|  | plasma-C2 | 0.94 | 0.22 | 0.66 | 0.74 | -0.43 |
|  | plasma-C3 | 0.05 | 0.97 | 0.01 | 0.99 | -0.02 |
|  | plasma-C8 | 1.32 | 0.08 | 1.09 | 0.11 | -3.24 |
|  | plasma-C10 | 1.58 | 0.03 | 1.53 | 0.27 | -1.91 |
|  | plasma-C16 | 2.19 | 0.00 | 4.57 | 0.44 | -1.18 |
|  | plasma-C18 | 2.02 | 0.00 | 3.15 | 0.50 | -1.00 |
|  | brain1-C0 | 0.91 | 0.23 | 0.64 | 1.38 | 0.47 |
|  | brain1-C2 | 0.09 | 0.87 | 0.06 | 1.04 | 0.06 |
|  | brain1-C3 | 1.26 | 0.09 | 1.04 | 0.01 | -6.71 |
|  | brain1-C4 | 1.01 | 0.19 | 0.71 | 0.91 | -0.14 |
|  | brain1-C8 | 0.78 | 0.30 | 0.52 | 0.62 | -0.70 |
|  | brain1-C10 | 1.20 | 0.11 | 0.97 | 0.67 | -0.58 |
|  | brain1-C16 | 1.31 | 0.10 | 1.01 | 1.42 | 0.51 |
|  | brain1-C18 | 0.67 | 0.43 | 0.37 | 1.27 | 0.35 |
|  | brain2-C0 | 0.51 | 0.38 | 0.41 | 1.41 | 0.50 |
|  | brain2-C2 | 0.61 | 0.66 | 0.18 | 0.88 | -0.19 |
|  | brain2-C3 | 1.67 | 0.01 | 2.06 | 0.02 | -5.68 |
|  | brain2-C4 | 0.79 | 0.23 | 0.63 | 0.98 | -0.03 |
|  | brain2-C8 | 0.59 | 0.33 | 0.49 | 1.28 | 0.35 |
|  | brain2-C10 | 0.56 | 0.32 | 0.49 | 0.98 | -0.03 |
|  | brain2-C16 | 0.15 | 0.80 | 0.09 | 1.06 | 0.09 |
|  | brain2-C18 | 0.29 | 0.52 | 0.29 | 1.29 | 0.36 |
|  | brain3-C0 | 0.83 | 0.25 | 0.60 | 1.15 | 0.21 |
|  | brain3-C2 | 1.00 | 0.18 | 0.75 | 1.30 | 0.38 |
|  | brain3-C3 | 1.52 | 0.03 | 1.52 | 0.02 | -5.70 |
|  | brain3-C4 | 0.78 | 0.32 | 0.49 | 1.02 | 0.02 |
|  | brain3-C8 | 0.89 | 0.27 | 0.57 | 1.27 | 0.34 |
|  | brain3-C10 | 0.16 | 0.79 | 0.10 | 1.00 | 0.00 |
|  | brain3-C16 | 0.60 | 0.51 | 0.29 | 0.80 | -0.32 |
|  | brain3-C18 | 0.37 | 0.69 | 0.16 | 0.78 | -0.37 |
|  | brain4-C0 | 1.00 | 0.18 | 0.74 | 1.27 | 0.34 |
|  | brain4-C2 | 0.50 | 0.46 | 0.34 | 1.17 | 0.22 |
|  | brain4-C3 | 1.22 | 0.10 | 1.00 | 0.02 | -5.95 |
|  | brain4-C4 | 0.63 | 0.40 | 0.40 | 0.97 | -0.04 |
|  | brain4-C8 | 0.96 | 0.20 | 0.69 | 0.86 | -0.21 |
|  | brain4-C10 | 1.12 | 0.15 | 0.81 | 0.70 | -0.51 |
|  | brain4-C16 | 0.43 | 0.66 | 0.18 | 1.11 | 0.15 |
|  | brain4-C18 | 0.29 | 0.76 | 0.12 | 1.08 | 0.11 |
| P2 | plasma-C0 | 1.43 | 0.07 | 1.18 | 0.32 | -1.66 |
|  | plasma-C2 | 1.85 | 0.01 | 1.90 | 0.39 | -1.36 |
|  | plasma-C3 | 1.83 | 0.01 | 1.88 | 0.16 | -2.66 |
|  | plasma-C8 | 1.32 | 0.11 | 0.95 | 0.10 | -3.38 |
|  | plasma-C10 | 1.78 | 0.02 | 1.76 | 0.10 | -3.34 |
|  | plasma-C16 | 0.19 | 0.84 | 0.08 | 0.95 | -0.07 |
|  | plasma-C18 | 0.52 | 0.53 | 0.28 | 1.13 | 0.18 |
|  | brain1-C0 | 0.53 | 0.53 | 0.28 | 0.91 | -0.14 |
|  | brain1-C2 | 1.13 | 0.16 | 0.80 | 1.48 | 0.56 |
|  | brain1-C3 | 0.71 | 0.40 | 0.40 | 0.48 | -1.05 |
|  | brain1-C4 | 0.27 | 0.74 | 0.13 | 0.98 | -0.03 |
|  | brain1-C8 | 0.54 | 0.51 | 0.29 | 0.78 | -0.36 |
|  | brain1-C10 | 1.64 | 0.03 | 1.58 | 0.40 | -1.31 |
|  | brain1-C16 | 0.81 | 0.33 | 0.48 | 1.37 | 0.45 |
|  | brain1-C18 | 0.19 | 0.83 | 0.08 | 1.09 | 0.13 |
|  | brain2-C0 | 0.33 | 0.71 | 0.15 | 1.17 | 0.23 |
|  | brain2-C2 | 0.23 | 0.78 | 0.11 | 0.93 | -0.11 |
|  | brain2-C3 | 1.03 | 0.20 | 0.70 | 0.41 | -1.29 |
|  | brain2-C4 | 0.40 | 0.67 | 0.17 | 1.01 | 0.02 |
|  | brain2-C8 | 0.84 | 0.32 | 0.49 | 1.28 | 0.35 |
|  | brain2-C10 | 0.87 | 0.26 | 0.59 | 0.98 | -0.03 |
|  | brain2-C16 | 0.57 | 0.49 | 0.31 | 1.47 | 0.56 |
|  | brain2-C18 | 0.67 | 0.43 | 0.37 | 1.66 | 0.73 |
|  | brain3-C0 | 0.49 | 0.57 | 0.24 | 0.93 | -0.10 |
|  | brain3-C2 | 1.33 | 0.09 | 1.02 | 1.43 | 0.51 |
|  | brain3-C3 | 0.39 | 0.64 | 0.19 | 0.72 | -0.48 |
|  | brain3-C4 | 2.03 | 0.00 | 2.63 | 1.09 | 0.12 |
|  | brain3-C8 | 0.93 | 0.26 | 0.59 | 1.27 | 0.35 |
|  | brain3-C10 | 0.10 | 0.90 | 0.04 | 1.00 | 0.00 |
|  | brain3-C16 | 0.79 | 0.34 | 0.47 | 0.71 | -0.50 |
|  | brain3-C18 | 0.31 | 0.74 | 0.13 | 0.81 | -0.31 |
|  | brain4-C0 | 1.54 | 0.07 | 1.18 | 0.85 | -0.23 |
|  | brain4-C2 | 1.58 | 0.04 | 1.40 | 1.57 | 0.65 |
|  | brain4-C3 | 0.01 | 1.00 | 0.00 | 1.00 | -0.01 |
|  | brain4-C4 | 0.84 | 0.30 | 0.52 | 1.06 | 0.09 |
|  | brain4-C8 | 1.00 | 0.23 | 0.64 | 0.86 | -0.22 |
|  | brain4-C10 | 1.01 | 0.22 | 0.66 | 0.83 | -0.27 |
|  | brain4-C16 | 0.02 | 0.96 | 0.02 | 0.99 | -0.02 |
|  | brain4-C18 | 0.02 | 0.98 | 0.01 | 0.99 | -0.01 |
| P3 | plasma-C0 | 0.46 | 0.55 | 0.26 | 1.25 | 0.32 |
|  | plasma-C2 | 1.52 | 0.01 | 1.95 | 0.51 | -0.98 |
|  | plasma-C3 | 0.90 | 0.19 | 0.72 | 0.61 | -0.72 |
|  | plasma-C8 | 0.90 | 0.20 | 0.69 | 0.33 | -1.61 |
|  | plasma-C10 | 1.57 | 0.01 | 1.86 | 0.23 | -2.11 |
|  | plasma-C16 | 1.03 | 0.12 | 0.91 | 0.73 | -0.46 |
|  | plasma-C18 | 1.19 | 0.09 | 1.06 | 0.81 | -0.30 |
|  | brain1-C0 | 1.41 | 0.03 | 1.57 | 2.23 | 1.16 |
|  | brain1-C2 | 1.51 | 0.02 | 1.77 | 1.87 | 0.90 |
|  | brain1-C3 | 1.34 | 0.04 | 1.41 | 3.76 | 1.91 |
|  | brain1-C4 | 0.77 | 0.21 | 0.69 | 0.92 | -0.12 |
|  | brain1-C8 | 0.10 | 0.77 | 0.11 | 0.92 | -0.11 |
|  | brain1-C10 | 1.06 | 0.08 | 1.11 | 0.79 | -0.34 |
|  | brain1-C16 | 1.22 | 0.10 | 1.01 | 1.41 | 0.50 |
|  | brain1-C18 | 1.43 | 0.04 | 1.45 | 1.75 | 0.81 |
|  | brain2-C0 | 0.98 | 0.18 | 0.74 | 1.79 | 0.84 |
|  | brain2-C2 | 0.27 | 0.69 | 0.16 | 1.11 | 0.15 |
|  | brain2-C3 | 0.93 | 0.21 | 0.68 | 3.82 | 1.93 |
|  | brain2-C4 | 0.56 | 0.39 | 0.40 | 0.99 | -0.02 |
|  | brain2-C8 | 0.73 | 0.29 | 0.53 | 1.27 | 0.34 |
|  | brain2-C10 | 0.85 | 0.23 | 0.65 | 0.98 | -0.03 |
|  | brain2-C16 | 0.18 | 0.86 | 0.06 | 1.05 | 0.07 |
|  | brain2-C18 | 0.88 | 0.23 | 0.64 | 1.56 | 0.64 |
|  | brain3-C0 | 0.80 | 0.27 | 0.58 | 5.32 | 2.41 |
|  | brain3-C2 | 1.29 | 0.04 | 1.43 | 2.20 | 1.14 |
|  | brain3-C3 | 1.47 | 0.03 | 1.47 | 12.28 | 3.62 |
|  | brain3-C4 | 0.86 | 0.19 | 0.73 | 1.03 | 0.04 |
|  | brain3-C8 | 0.85 | 0.26 | 0.59 | 1.25 | 0.32 |
|  | brain3-C10 | 0.45 | 0.63 | 0.20 | 1.00 | -0.01 |
|  | brain3-C16 | 0.35 | 0.65 | 0.19 | 1.18 | 0.24 |
|  | brain3-C18 | 0.54 | 0.46 | 0.34 | 1.52 | 0.61 |
|  | brain4-C0 | 1.18 | 0.03 | 1.50 | 1.57 | 0.65 |
|  | brain4-C2 | 1.26 | 0.03 | 1.57 | 1.53 | 0.62 |
|  | brain4-C3 | 1.43 | 0.01 | 1.95 | 5.70 | 2.51 |
|  | brain4-C4 | 0.37 | 0.70 | 0.16 | 0.99 | -0.02 |
|  | brain4-C8 | 0.87 | 0.22 | 0.65 | 0.87 | -0.20 |
|  | brain4-C10 | 0.95 | 0.19 | 0.73 | 0.84 | -0.26 |
|  | brain4-C16 | 0.71 | 0.38 | 0.43 | 0.76 | -0.39 |
|  | brain4-C18 | 0.66 | 0.49 | 0.31 | 0.78 | -0.36 |
| P4 | plasma-C0 | 0.14 | 0.86 | 0.06 | 0.92 | -0.13 |
|  | plasma-C2 | 1.64 | 0.02 | 1.77 | 0.45 | -1.15 |
|  | plasma-C3 | 1.57 | 0.02 | 1.61 | 0.28 | -1.86 |
|  | plasma-C8 | 1.27 | 0.08 | 1.08 | 0.11 | -3.20 |
|  | plasma-C10 | 1.77 | 0.01 | 2.16 | 0.06 | -4.12 |
|  | plasma-C16 | 1.41 | 0.05 | 1.31 | 0.77 | -0.38 |
|  | plasma-C18 | 0.55 | 0.48 | 0.32 | 0.90 | -0.14 |
|  | brain1-C0 | 1.35 | 0.06 | 1.20 | 1.49 | 0.57 |
|  | brain1-C2 | 0.53 | 0.50 | 0.30 | 1.18 | 0.24 |
|  | brain1-C3 | 0.56 | 0.48 | 0.32 | 1.45 | 0.54 |
|  | brain1-C4 | 0.95 | 0.22 | 0.67 | 0.91 | -0.13 |
|  | brain1-C8 | 0.20 | 0.81 | 0.09 | 0.93 | -0.10 |
|  | brain1-C10 | 1.19 | 0.11 | 0.95 | 0.80 | -0.33 |
|  | brain1-C16 | 0.89 | 0.25 | 0.60 | 1.39 | 0.48 |
|  | brain1-C18 | 0.82 | 0.30 | 0.52 | 1.49 | 0.58 |
|  | brain2-C0 | 0.82 | 0.33 | 0.48 | 1.15 | 0.20 |
|  | brain2-C2 | 1.64 | 0.05 | 1.30 | 0.60 | -0.73 |
|  | brain2-C3 | 1.80 | 0.01 | 1.95 | 3.45 | 1.79 |
|  | brain2-C4 | 0.02 | 0.77 | 0.11 | 0.99 | -0.01 |
|  | brain2-C8 | 0.74 | 0.30 | 0.52 | 1.29 | 0.37 |
|  | brain2-C10 | 0.59 | 0.33 | 0.49 | 0.98 | -0.03 |
|  | brain2-C16 | 1.15 | 0.23 | 0.63 | 0.69 | -0.53 |
|  | brain2-C18 | 0.84 | 0.34 | 0.47 | 0.73 | -0.46 |
|  | brain3-C0 | 1.12 | 0.14 | 0.85 | 1.34 | 0.42 |
|  | brain3-C2 | 0.63 | 0.42 | 0.38 | 1.19 | 0.25 |
|  | brain3-C3 | 1.38 | 0.06 | 1.25 | 4.77 | 2.25 |
|  | brain3-C4 | 0.91 | 0.24 | 0.62 | 1.02 | 0.04 |
|  | brain3-C8 | 0.78 | 0.31 | 0.51 | 1.24 | 0.31 |
|  | brain3-C10 | 0.95 | 0.22 | 0.67 | 0.99 | -0.01 |
|  | brain3-C16 | 0.53 | 0.51 | 0.29 | 0.80 | -0.33 |
|  | brain3-C18 | 0.17 | 0.83 | 0.08 | 1.13 | 0.18 |
|  | brain4-C0 | 0.53 | 0.51 | 0.29 | 1.22 | 0.28 |
|  | brain4-C2 | 0.58 | 0.46 | 0.34 | 0.82 | -0.28 |
|  | brain4-C3 | 0.85 | 0.28 | 0.56 | 4.51 | 2.17 |
|  | brain4-C4 | 1.08 | 0.15 | 0.82 | 0.95 | -0.07 |
|  | brain4-C8 | 1.03 | 0.18 | 0.76 | 0.85 | -0.23 |
|  | brain4-C10 | 1.03 | 0.18 | 0.75 | 0.83 | -0.26 |
|  | brain4-C16 | 0.24 | 0.78 | 0.11 | 0.94 | -0.10 |
|  | brain4-C18 | 0.35 | 0.66 | 0.18 | 1.10 | 0.14 |

1. **The values of VIP, -Log_10_ *P*-value and Log_2_ Fold Change in the OPLS-DA model (second strategy)**

**Table S11** The values of VIP, -Log_10_ *P*-value and Log_2_ Fold Change in the OPLS-DA model of second strategy.

| Sample ID | VIP | *P*-value | -Log_10_ *P*-value | Fold Change | Log_2_ Fold Change |
| --- | --- | --- | --- | --- | --- |
| plasma-C0 | 0.19 | 0.74 | 0.13 | 0.88 | -0.19 |
| plasma-C2 | 2.11 | 0.00 | 3.39 | 0.46 | -1.14 |
| plasma-C3 | 1.75 | 0.01 | 2.26 | 0.37 | -1.43 |
| plasma-C8 | 1.53 | 0.01 | 1.85 | 0.19 | -2.40 |
| plasma-C10 | 2.31 | 0.00 | 4.67 | 0.14 | -2.87 |
| plasma-C16 | 0.84 | 0.23 | 0.64 | 0.80 | -0.32 |
| plasma-C18 | 0.37 | 0.62 | 0.21 | 0.93 | -0.10 |
| brain1-C0 | 0.86 | 0.16 | 0.79 | 1.58 | 0.66 |
| brain1-C2 | 1.18 | 0.06 | 1.21 | 1.53 | 0.61 |
| brain1-C3 | 0.66 | 0.31 | 0.51 | 2.00 | 1.00 |
| brain1-C4 | 0.99 | 0.11 | 0.94 | 0.94 | -0.10 |
| brain1-C8 | 0.42 | 0.50 | 0.30 | 0.88 | -0.18 |
| brain1-C10 | 1.36 | 0.03 | 1.46 | 0.67 | -0.58 |
| brain1-C16 | 1.06 | 0.12 | 0.93 | 1.39 | 0.48 |
| brain1-C18 | 0.86 | 0.19 | 0.72 | 1.46 | 0.55 |
| brain2-C0 | 0.68 | 0.36 | 0.44 | 1.39 | 0.48 |
| brain2-C2 | 0.39 | 0.62 | 0.21 | 0.89 | -0.16 |
| brain2-C3 | 0.77 | 0.29 | 0.54 | 2.62 | 1.39 |
| brain2-C4 | 0.21 | 0.86 | 0.07 | 1.00 | 0.00 |
| brain2-C8 | 1.34 | 0.06 | 1.24 | 1.28 | 0.35 |
| brain2-C10 | 1.20 | 0.04 | 1.36 | 0.98 | -0.03 |
| brain2-C16 | 0.05 | 0.86 | 0.06 | 1.07 | 0.10 |
| brain2-C18 | 0.39 | 0.55 | 0.26 | 1.33 | 0.41 |
| brain3-C0 | 0.49 | 0.48 | 0.32 | 2.68 | 1.42 |
| brain3-C2 | 1.06 | 0.10 | 0.99 | 1.64 | 0.71 |
| brain3-C3 | 0.91 | 0.17 | 0.77 | 5.93 | 2.57 |
| brain3-C4 | 1.24 | 0.05 | 1.34 | 1.05 | 0.06 |
| brain3-C8 | 1.31 | 0.05 | 1.34 | 1.25 | 0.33 |
| brain3-C10 | 0.47 | 0.43 | 0.36 | 1.00 | -0.01 |
| brain3-C16 | 0.20 | 0.73 | 0.13 | 0.91 | -0.14 |
| brain3-C18 | 0.22 | 0.71 | 0.15 | 1.17 | 0.23 |
| brain4-C0 | 0.51 | 0.37 | 0.43 | 1.23 | 0.30 |
| brain4-C2 | 0.68 | 0.25 | 0.60 | 1.29 | 0.37 |
| brain4-C3 | 0.86 | 0.18 | 0.74 | 3.89 | 1.96 |
| brain4-C4 | 0.00 | 0.95 | 0.02 | 1.00 | 0.00 |
| brain4-C8 | 1.30 | 0.02 | 1.65 | 0.86 | -0.22 |
| brain4-C10 | 1.34 | 0.02 | 1.75 | 0.83 | -0.26 |
| brain4-C16 | 0.36 | 0.56 | 0.25 | 0.89 | -0.17 |
| brain4-C18 | 0.12 | 0.84 | 0.08 | 0.95 | -0.07 |

1. **The detailed AUC values of potential biomarkers in first and second strategies**

**Table S12** The AUC values of potential biomarkers.

| Strategy | Group | Potential Biomarker | AUC |
| --- | --- | --- | --- |
| First strategy | P1 | plasma-C10 | 0.875 |
|  |  | plasma-C16 | 1.000 |
|  |  | plasma-C18 | 1.000 |
|  |  | brain2-C2 | 0.875 |
|  |  | brain3-C3 | 1.000 |
|  | P2 | plasma-C2 | 0.920 |
|  |  | plasma-C3 | 0.840 |
|  |  | plasma-C10 | 1.000 |
|  |  | brain1-C10 | 0.920 |
|  |  | brain3-C4 | 0.040 |
|  |  | brain4-C2 | 0.120 |
|  | P3 | plasma-C2 | 0.920 |
|  |  | plasma-C10 | 0.920 |
|  |  | brain1-C0 | 0.000 |
|  |  | brain1-C2 | 0.080 |
|  |  | brain1-C3 | 0.080 |
|  |  | brain1-C18 | 0.200 |
|  |  | brain3-C2 | 0.080 |
|  |  | brain3-C3 | 0.000 |
|  |  | brain4-C0 | 0.040 |
|  |  | brain4-C2 | 0.160 |
|  |  | brain4-C3 | 0.080 |
|  | P4 | plasma-C2 | 0.875 |
|  |  | plasma-C3 | 0.792 |
|  |  | plasma-C10 | 1.000 |
|  |  | plasma-C16 | 0.833 |
|  |  | brain2-C3 | 0.000 |
| Second strategy | Pcom | plasma-C2 | 0.897 |
|  |  | plasma-C3 | 0.765 |
|  |  | plasma-C8 | 0.824 |
|  |  | plasma-C10 | 0.971 |
|  |  | brain1-C10 | 0.765 |
|  |  | brain2-C8 | 0.529 |
|  |  | brain2-C10 | 0.500 |
|  |  | brain3-C4 | 0.206 |
|  |  | brain3-C8 | 0.485 |
|  |  | brain4-C8 | 0.926 |
|  |  | brain4-C10 | 0.824 |
